# The Validation of Nematode-Specific Acetylcholine-Gated Chloride Channels as Potential Anthelmintic Drug Targets

**DOI:** 10.1371/journal.pone.0138804

**Published:** 2015-09-22

**Authors:** Claudia M. Wever, Danielle Farrington, Joseph A. Dent

**Affiliations:** Department of Biology, McGill University, Montreal, Quebec, Canada; New England Biolabs, UNITED STATES

## Abstract

New compounds are needed to treat parasitic nematode infections in humans, livestock and plants. Small molecule anthelmintics are the primary means of nematode parasite control in animals; however, widespread resistance to the currently available drug classes means control will be impossible without the introduction of new compounds. Adverse environmental effects associated with nematocides used to control plant parasitic species are also motivating the search for safer, more effective compounds. Discovery of new anthelmintic drugs in particular has been a serious challenge due to the difficulty of obtaining and culturing target parasites for high-throughput screens and the lack of functional genomic techniques to validate potential drug targets in these pathogens. We present here a novel strategy for target validation that employs the free-living nematode *Caenorhabditis elegans* to demonstrate the value of new ligand-gated ion channels as targets for anthelmintic discovery. Many successful anthelmintics, including ivermectin, levamisole and monepantel, are agonists of pentameric ligand-gated ion channels, suggesting that the unexploited pentameric ion channels encoded in parasite genomes may be suitable drug targets. We validated five members of the nematode-specific family of acetylcholine-gated chloride channels as targets of agonists with anthelmintic properties by ectopically expressing an ivermectin-gated chloride channel, AVR-15, in tissues that endogenously express the acetylcholine-gated chloride channels and using the effects of ivermectin to predict the effects of an acetylcholine-gated chloride channel agonist. In principle, our strategy can be applied to validate any ion channel as a putative anti-parasitic drug target.

## Introduction

Nematode parasites are a major source of disease in both humans and livestock and are a significant crop pest. According to a 2014 report from the World Health Organization, over 1.5 billion people are infected with nematode parasites worldwide [1]. Nematode parasites also devastate crops across the globe [2], and the majority of cattle and sheep farms around the world are plagued by nematode parasites [3]. While available anthelmintic drugs have been successful in controlling animal parasites, their continued effectiveness is threatened by the evolution of drug resistance [3–7]. Controlling resistant nematode parasites with currently available anthelmintic drugs has become challenging, which highlights the need for continuous development of new compounds that act on novel targets so as to avoid receptor-mediated mechanisms of cross-resistance.

Many anthelmintic drugs have been identified in whole organism screens of compound libraries using death, paralysis, or developmental arrest as endpoints [8–10]. These primary screens require extensive secondary screens to identify the subset of compounds: 1) with novel targets and thus not subject to cross-resistance with existing drugs, and 2) with no adverse off-target effects. An alternative strategy is to first identify protein targets that have desirable characteristics and subsequently screen for drugs that specifically act on these targets, a strategy that has been the mainstay of industry drug discovery for over 20 years. Mechanism-based screening is particularly challenging for parasitic nematodes due to difficulties in culturing parasitic species and our current inability to manipulate parasitic genomes and transcriptomes with available technological platforms.

Among the criteria for an ideal anthelmintic drug target are: 1) that it be absent from host organisms, 2) that it is not the target of an existing compound and therefore not subject to cross-resistance with existing drugs, 3) that it belongs to a multi-gene family which would increase the likelihood of identifying a drug with multiple targets, thereby potentially increasing the efficacy and possibly slowing the evolution of resistance, and 4) that the target is essential to the life-cycle of the organism. The first three criteria can be evaluated using bioinformatics and available genome databases [11–22]. However, identifying targets essential to overall fitness of the target species requires experimental validation.

Genetic strategies have the potential to validate a target *in vivo* [23–26]. If the goal were to identify an antagonist/blocker of the target, the phenotype of a corresponding loss-of-function or knockout mutation of the target would reflect the physiological response to an antagonist. In some instances, however, phenotypes may only be deleterious with an agonist or modulator of the target activity, in which case a hypermorphic or gain-of-function mutation would better reflect the drug response. Ideally, the gain-of-function should be inducible so that dominant lethal phenotypes and phenotypes at different stages of development can be tested. The difficulty of identifying a mutation that produces the desired hypermorphic or gain-of-function effect is a significant obstacle to success in the target-based approach and we currently lack other functional genomic tools to allow us to predict the effects of agonist drugs.

The problem of validating targets for which an agonist is required is particularly acute in the case of anthelmintics. Most available anthelmintics, such as levamisole, pyrantel, ivermectin, and monepantel, are agonists that activate members of the pentameric ligand-gated ion channel (pLGIC) superfamily [27]. The superfamily of pLGICs in nematodes is large, diverse, and comprises many channel subunits that are specific to invertebrates or to the phylum nematoda [28]. Among these are the acetylcholine-gated chloride (ACC) channels. The ACC channel clade was first identified in *C*. *elegans* and comprises eight subunit genes: *acc-1*, *acc-2*, *acc-3*, *acc-4*, *lgc-46*, *lgc-47*, *lgc-48*, and *lgc-49* [29]. Features of the ACC channel family that make its members attractive anthelmintic drug targets include: 1) the ACCs appear to be nematode-specific, 2) the ACCs are not targets of current anthelmintics, 3) though they bind acetylcholine, their pharmacology is distinct from nicotinic-type receptors, including the nematode levamisole receptors, and 4) the eight subunit genes suggest a multichannel, and thus multi-target, family [29]. It remains only to verify that a drug acting on the ACCs would be sufficiently toxic to qualify as an anthelmintic compound.

Here, we take a genetic approach to the validation of the ACC channels as suitable anthelmintic drug targets. By analyzing the phenotypes of ACC knockouts, which should mimic the effects of ACC channel antagonists, we show that antagonists of these channels would likely not be sufficiently deleterious to the health or behavior of the worm to be suitable anthelmintics. By contrast, ivermectin-induced activation of ectopically expressed chloride channels (ivermectin receptors) in ACC-expressing tissues, which should mimic the effect of an ACC channel agonist, resulted in paralysis and developmental arrest. We therefore conclude ACC channels would be suitable targets for anthelmintic channel agonists. This strategy, which has been previously used to evaluate the behavioral effects of inhibiting neural circuits [30], has general utility for validating agonists of ion channels as effective anthelmintics.

## Methods

### Ethics Statement

We have used *Xenopus laevis* oocytes to express ion channels. Tricaine methanesulfonate (MS222) was used an anesthetic administered to the frogs for oocyte extraction. Frogs were monitored daily for two weeks for appetite as well as for any complications such as dehiscence or infection of the surgical site. All animal care protocols are in compliance with McGill's animal care Standard Operating Procedures; protocol number 2006–5284. All animal care procedures were approved by the McGill Facility Animal Care Committee (FACC).

### Phylogeny

**Fig 1:**
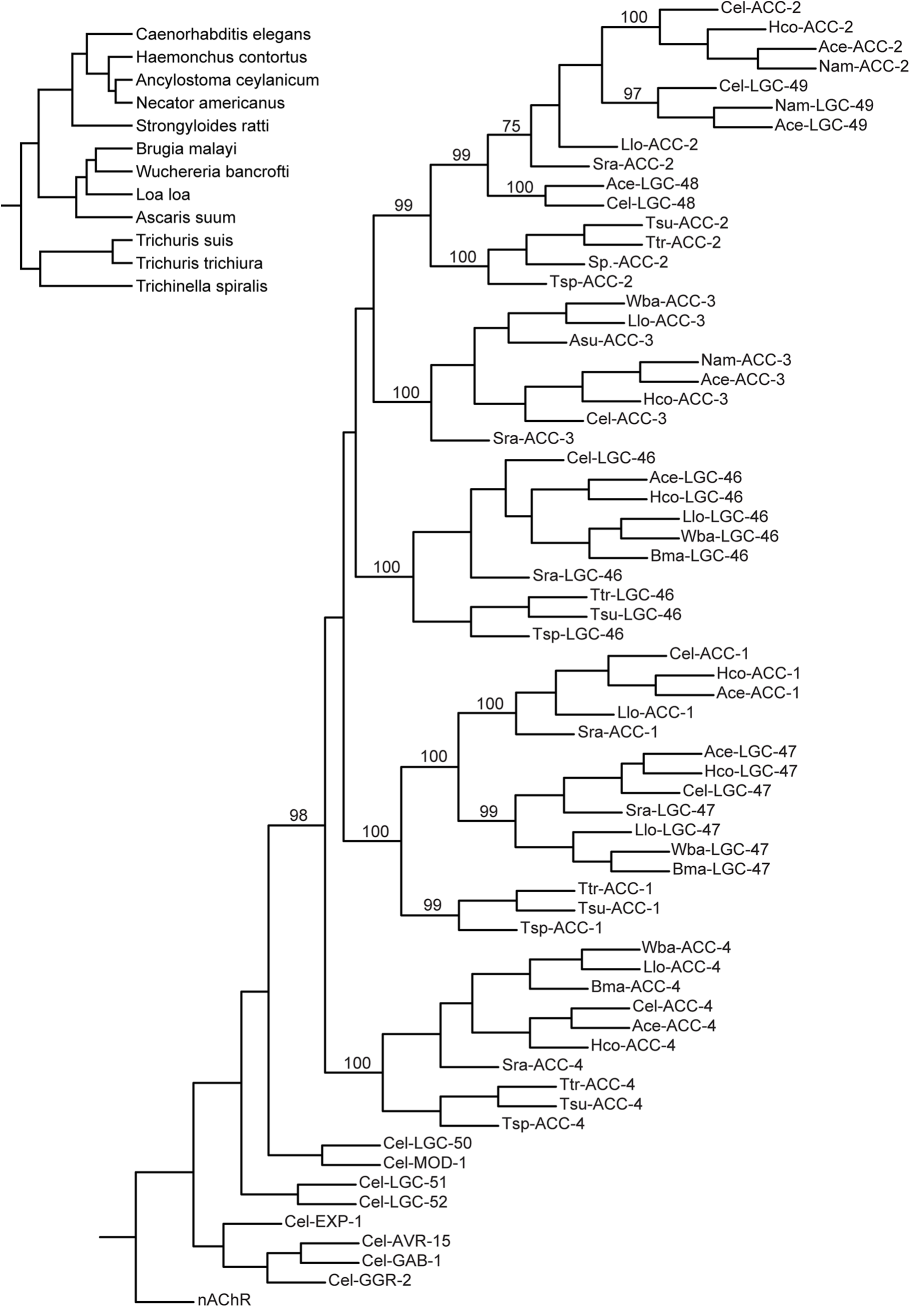
Phylogenetic tree of ACCs in *C*. *elegans* and parasitic nematode species. A maximum likelihood tree made from predicted protein sequences identified using *C*. *elegans* (cel) ACCs as BLAST queries for similar sequences in *Haemonchus contortus* (Hco), *Ancylostoma ceylanicum* (Ace), *Necator americanus* (Nam), *Strongyloides ratti* (Sra), *Brugia malayi* (Bma), *Wuchereria bancrofti* (Wba), *Loa loa* (Llo), *Ascaris suum* (Asu), *Trichuris suis* (Tsu), *Trichuris trichiura* (Ttr), and *Trichinella spiralis* (Tsp). Bootstrap values out of 100 are indicated at ACC clade-defining branches. The tree was rooted to the α1 subunit of the torpedo nicotinic AChR [81]. Representative subunits from other *C*. *elegans* chloride-selective pLGIC clades (LGC-50, LGC-51, LGC-52, MOD-1, EXP-1, AVR-15, GAB-1, and GGR-2) were also included to ensure that predicted ACC orthologs from other species grouped with the celACCs instead of other *C*. *elegans* chloride channels. Inset: Phylogenetic relationship of nematodes species. Branch lengths are approximate.

*C*. *elegans* ACC protein sequences were used to query the NCBI database (http://blast.ncbi.nlm.nih.gov/) of nematode sequences. The orthologous sequences from parasites (S1 Table) were aligned (ClustalW), GeneDoc was used to create a percent homology table (Table 1) and PhyML 3.0 [31] was used to create the phylogenetic tree with 100 bootstrap repetitions (Fig 1). We named the ACC homologues according to Beech et al. [32] based on the resulting tree.

**Table 1:** ACC orthologs in parasitic nematodes.

Organism	ACC-1	LGC-47	ACC-3	ACC-4	LGC-46	LGC-48	ACC-2	LGC-49
*Haemonchus contortus*	70%	57%	65%	76%	72%		67%	
*Ancylostoma ceylanicum*	53%	68%	67%	79%	74%	50%	61%	70%
*Necator americanus*			61%				44%	50%
*Strongyloides ratti*	63%	52%	52%	52%	50%	56%		
*Brugia malayi*		51%		45%	63%			
*Wuchereria bancrofti*		46%	32%	34%	62%			
*Loa loa*	55%	51%	48%	60%	54%	45%		
*Ascaris suum*			51%					
*Trichuris suis*	40%			53%	49%	45%		
*Trichuris trichiura*	42%			53%	48%	45%		
*Trichinella spiralis*	39%			23%	49%	41%		

The *C*. *elegans* ACC genes are represented as columns; a value in the corresponding cell indicates the presence of an ortholog of that gene in a specific species, with the percent homology indicated. Merged cells indicate that the parasitic gene is an ortholog of an ancestral gene that gave rise to multiple ACCs in *C*. *elegans;* the highest corresponding percent homology is indicated in the table.

### Plasmids

We used PCR to amplify regions of the ACC promoters from *C*. *elegans*. The specific primers used are listed in S2 Table. We made operon constructs of the different ACC promoters with an SL2 trans-splicing domain from the *gpd* gene operon between the promoter and the AVR-15 open reading frame tagged with YFP in the intracellular loop of the subunit. A modified pPD49.26 vector (Fire Vector Kit, Addgene) was used as a starting point, which has an SL2 transplicing domain cloned into the multiple cloning site along with the AVR-15::YFP. This vector was then cut with BamHI and MscI (New England Biolabs) and the promoters for the ACC genes were cloned into the vector using the Infusion cloning system (Clontech).

### Oocyte Expression and Electrophysiology

PCR-amplified GFP variants with Kpn I sites encoded in the primers were cloned into the Kpn I site of AVR-15 cDNA constructs in the pT7 vector [33]. The vectors were linearized with BamHI to synthesize cRNA using the mMESSAGE mMACHINE T7 kit (Ambion) for injection into oocytes.

Oocytes were harvested according to McGill Standard Operating Procedures for amphibian surgery. Oocytes were kept in standard ND96 solution unless otherwise noted and the solution was changed at least once a day while the oocytes were in culture. Oocytes were injected with 25ng of *in vitro* synthesized RNA (50nL at 500ng/μl). Injections were performed using the Nanoject system (Drummond Scientific, Broomall, PA) and injected oocytes were incubated at 15°C for approximately 40h before measurements were taken.

Unless otherwise indicated all Two-Electrode Voltage Clamp (TEVC) experiments were performed using a Maltese Cross chamber (ALA Scientific Instruments, Westbury, NY) as described in Purtrenko et al. [29]

Recordings were done using the AxoClamp 2B amplifier (Axon Instruments, Foster City, CA). Data were acquired at 1 kHz using the Clampex 8.1 software (Axon Instruments, Foster City, CA) and analyzed using the Clampfit 8.1 software (Axon Instruments, Foster City, CA). Hill curves were fit to the concentration-response data using the IGOR Pro 6.0.2.4 Software (Wavemetrics Inc., Portland, OR).

### *C*. *elegans* Strains

A complete list of strains used is found in S4 Table. The Δ*acc-1* deletion strain carries the allele *tm3268*. This knockout was generated by the National BioResource Project (Tokyo, Japan), which is part of the International *C*. *elegans* Gene Knockout Consortium. The Δ*acc-2* strain VC1757 acc-2(*ok2216*) and the Δ*lgc-49* strain VC40013 *lgc-49(gk246966)* were made by the *C*. *elegans* Reverse Genetics Core Facility at the University of British Columbia, which is part of the international *C*. *elegans* Gene Knockout Consortium. The Δ*lgc-47* strain RB2187 *lgc-47(ok296)* was made by the *C*. *elegans* Gene Knockout Project at OMRF, which is part of the international *C*. *elegans* Gene Knockout Consortium. These strains were outcrossed at least seven times to wild type N2 worms and the respective deletions were confirmed using primers described in S3 Table. S1 Fig illustrates the *acc* gene deletions, which we predict to be loss-of-functions alleles.

All of the *Pacc*::AVR-15::YFP strains were made by injecting plasmids into JD369 (*avr-14*, *avr-15*, *glc-1*, *glc-3)* worms. Constructs were microinjected into worms along with a transformation marker, *rol-6*, as described by Mello et al. [34]. All ACC promoter constructs were injected at concentrations ranging from 20-50ng/μL DNA in water. Progeny of the injected animals were screened for the roller phenotype and singled 2–3 independent stable (F2) strains were kept for each *Pacc*:: AVR-15::YFP strain and the extrachromosomal arrays were integrated into the genome by gamma-irradiation [35].

### Ivermectin Assays

Worm culture dishes were made from standard NGM media [36]. Before pouring the plates, IVM dissolved in DMSO was added to the molten agar mixture to a final concentration of DMSO of 1%. IVM concentrations varied from 0.1ng/mL to 500ng/mL in the agar NGM mixture, with low IVM concentrations acting as control conditions due to our need to take the log of the smallest value for our analysis. All plates were seeded with HB101 bacteria.

*C*. *elegans* eggs were harvested by standard alkaline-bleach techniques [37] and the eggs were plated by pipetting a fixed volume onto IVM plates seeded with HB101 bacteria. The plates were left at room temperature for 3 days and worms that had developed beyond the first larval stage (L1) were scored as surviving. Each IVM concentration was tested in triplicate for each strain. All results are given as percent survival, averaged over experiments, and normalized to the plate with the greatest number of survivors. For assays using adults, eggs were placed on NGM plates with HB101 bacteria for two days and then transferred to IVM plates, also seeded with HB101, as adults. Worms were scored as dead when they were no longer moving, their pharynxes were not pumping and they did not respond to gentle prodding with a platinum worm pick [38].

### Data Analysis

For each transgene, two transgenic strains representing independent genome integration events were tested (see S4 Table for complete strain list) and the resulting data was averaged to calculate EC_50_, paralysis or survival rate for each *acc* promoter::AVR-15 fusion combination. Hill curves were fit to the IVM L1 escape data using the IGOR Pro 6.0.2.4 Software (Wavemetrics Inc., Portland, OR) to obtain EC_50_ values. The base and maximum values were manually set to 0 and 1, respectively. Statistical analysis of the various *acc* mutant phenotypes was done in SigmaPlot 12.0 (Systat Software Inc.). A t-test was used to compare N2 values to the mutant strain values.

## Results

### ACC Phylogeny

To determine whether drugs targeting ACCs could be broad-spectrum anthelmintics within the nematode clade, we conducted BLAST searches of available nematode genomes using *C*. *elegans* ACC protein sequences as queries. The available genomes represent the major nematode clades and reflect the diversity of the nematode phylum [39]. To identify ACC orthologs, a phylogenetic tree was constructed (Fig 1). The ACCs are highly conserved across nematode species, as summarized in Table 1, with orthologs of most *C*. *elegans* ACC subunits in many nematode species. We did not find any ACCs in the *Onchocerca volvulus* or *Onchocerca ochengi* genomes in their current state of completion. S1 Table lists the genes used in the phylogenetic analysis, and includes new ACC names for all of the genes, using nomenclature as suggested by Beech et al. [32] From these results, we conclude that a drug targeting the ACCs would likely affect most nematode species.

### Antagonists of ACCs would likely not be potent anthelmintics

**Fig 2:**
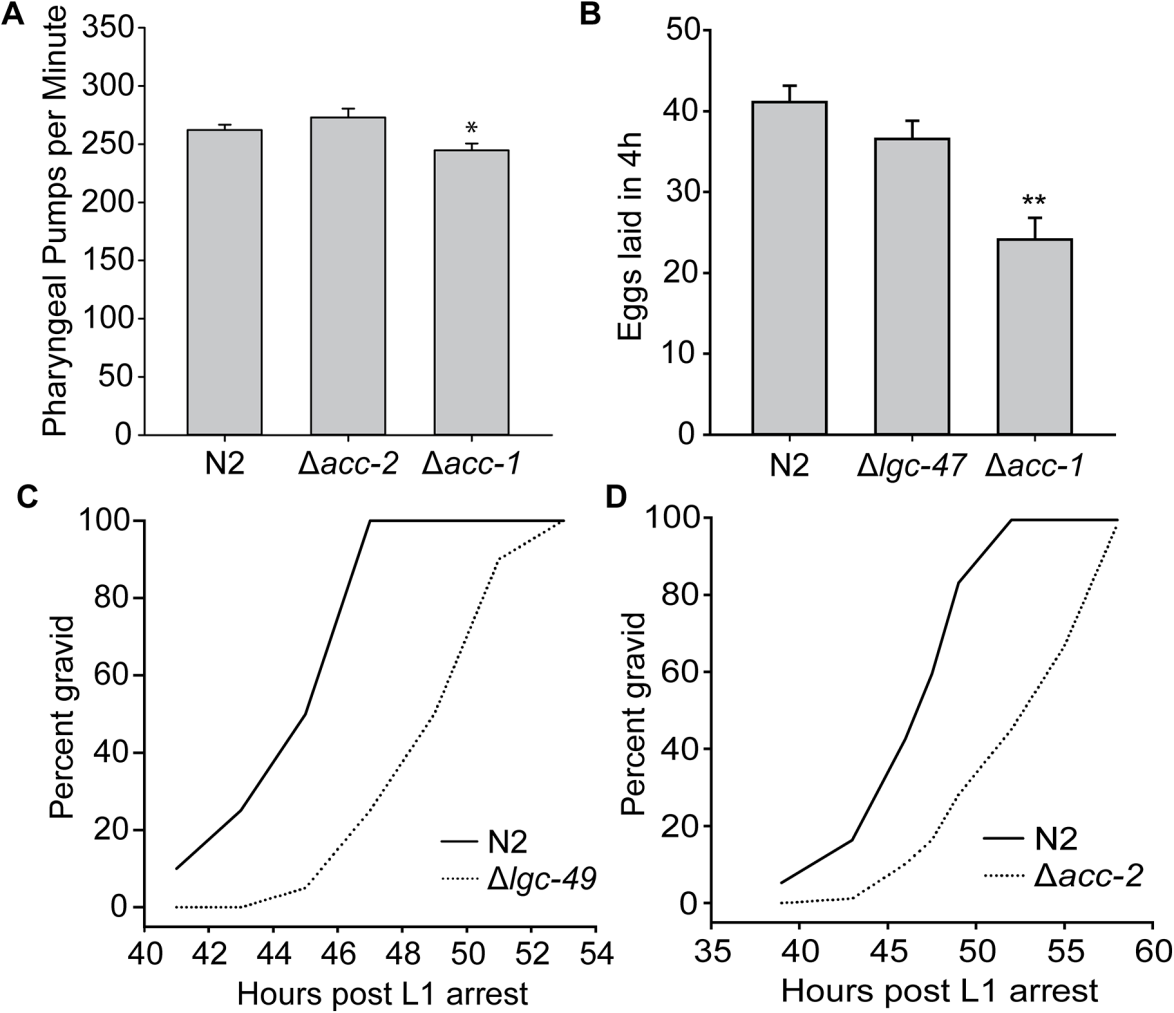
Deletions in various *acc* genes result in subtle phenotypes. **(A)** Worms with deletions in *acc-1* (tm3268) but not *acc-2* (ok2216) have slightly slowed pharyngeal pumping rates; error bars ± 1SEM, * indicates p<0.05. **(B)** Worms with deletions in *acc-1* (tm3268) but not *lgc-47* (ok2963) have decreased egg-laying; error bars ± 1SEM, ** indicates p<0.001. **(C, D)** Worms with deletions in *lgc-49* (gk246966) and *acc-2* (ok2216) have slightly slowed development when compared to wildtype N2.

To understand the likely effects of an ACC channel antagonist, we examined strains of *C*. *elegans* with mutations in four of the eight ACC genes: *acc-1*, *acc-2*, *lgc-47*, and *lgc-49*. All mutant worms were viable and fertile. We investigated three phenotypes that are characteristic of existing anthelmintic drug classes in more detail: development (benzimidazoles, levamisole, macrocyclic lactones), pharyngeal pumping (macrocyclic lactones) and egg-laying (macrocyclic lactones) [40,41]. *acc-1* but not *acc-2* mutant worms showed slightly slower pharyngeal pumping rates, 244.8 ± 5.8 and 273.1 ± 7.4 pumps per minute respectively, when compared to N2 with 262.2 ± 4.6 pumps per minute (Fig 2A). *acc-1* but not *lgc-47* mutant worms also showed significantly decreased egg-laying frequency compared to N2, with *acc-1* laying 24.2 ± 2.7 eggs in 4h, *lgc-47* laying 36.6 ± 2.3 eggs in 4h, compared to 41.1 ± 2.1 eggs laid in 4h for N2 (Fig 2B). *acc-2* and *lgc-49* mutants had slightly slowed development compared to N2 (Fig 2C and 2D). None of the phenotypes associated with mutations in these *acc* genes were severe enough to be substantially detrimental to the overall health or fertility of the worm; therefore we conclude that an ACC antagonist would likely not be an effective anthelmintic.

### ACC Agonists would make potent anthelmintics

**Fig 3:**
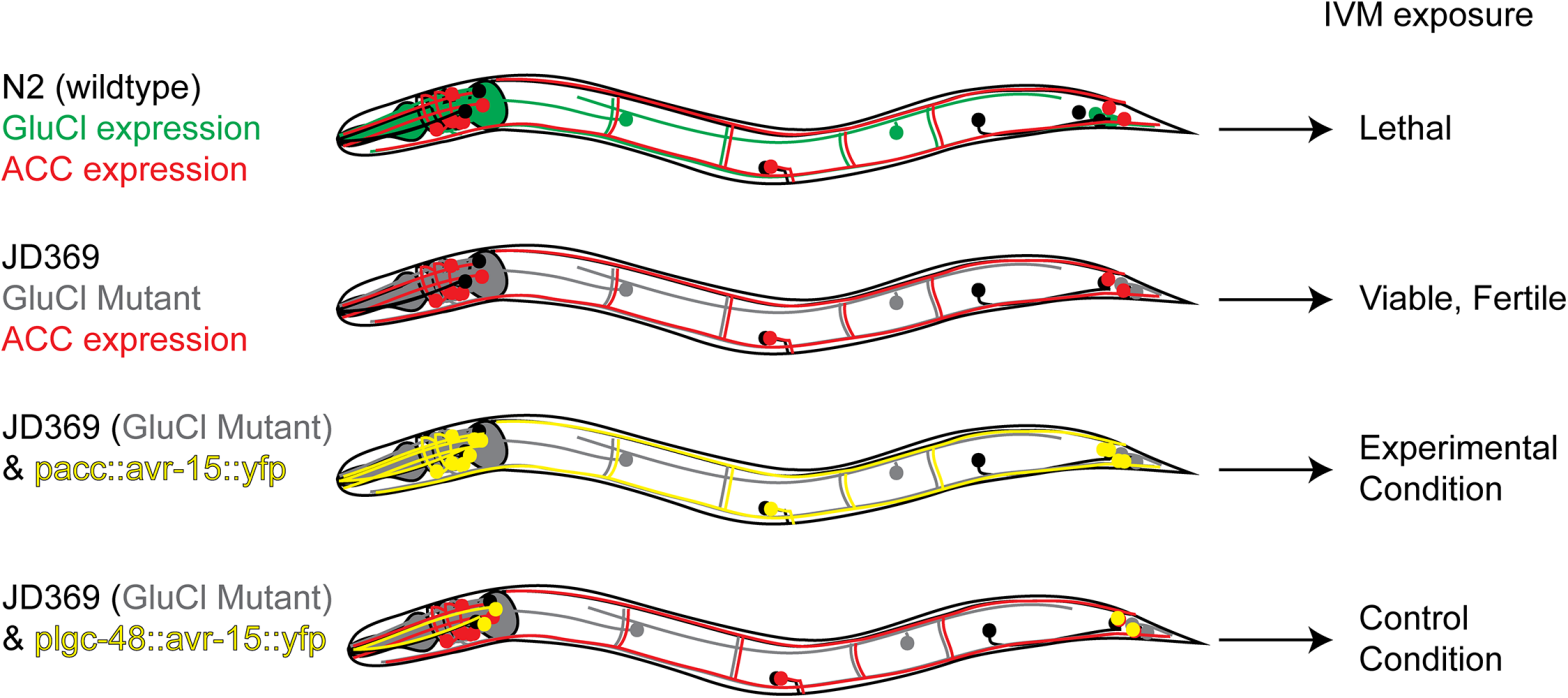
Schematic representation of our strategy to validate the ACCs as targets of agonist drugs. Wild-type *C*. *elegans* express the IVM-targeted GluCls in essential tissues and thus are sensitive to IVM. The strain JD369 lacks functional IVM-targeted GluCl channels, and is thus insensitive to IVM. By selectively reintroducing the GluCl channel subunit AVR-15 under the control of the different ACC promoters, we are able to generate strains that have IVM-gated channels exclusively in tissues that endogenously express the ACC channels. We can then treat these strains with IVM to predict the effects of a direct ACC agonist. Green neurons represent endogenous GluCl-expressing neurons. Red neurons indicate ACC-expressing neurons. Grey neurons indicate loss of GluCl expression in endogenous cells. Yellow neurons indicate GluCl AVR-15::YFP transgene expressed in ACC-expressing cells.

Most existing anthelmintics that act on pLGICs are agonists (e.g. levamisole, macrocyclic lactones, monepantel). We therefore wanted to determine if agonists of ACC channels could be expected to exhibit toxicity to nematodes. We reasoned that an ACC agonist would have the same effect on the cells that express them as an agonist of the structurally similar glutamate-gated chloride channels (GluCls) ectopically expressed in those same cells (Fig 3). Ivermectin is an pseudo-irreversible agonist of the GluCls, which are the physiologically relevant targets of its nematocidal action [33,42,43]. The binding of ivermectin to GluCls in the muscles and nervous system generates permanently shunting chloride currents, which result in tissues that are refractory to excitation. By expressing the GluCl subunit AVR-15 under the control of various ACC promoters and treating the resulting transgenic animals with IVM, we can determine the effects of over-inhibiting the ACC-expressing tissues and should be able to estimate the potential toxicity of a corresponding ACC agonist. Although any inducible chloride channel could in principle be used, the properties of AVR-15 are well suited to our experimental approach: it forms a homomeric ivermectin-gated channel, it expresses ectopically in *C*. *elegans*, ivermectin is permeable to the *C*. *elegans* cuticle and AVR-15 belongs to the same pentameric receptor superfamily [33] as the ACCs and therefore would be expected to show similar cell biological properties.

**Fig 4:**
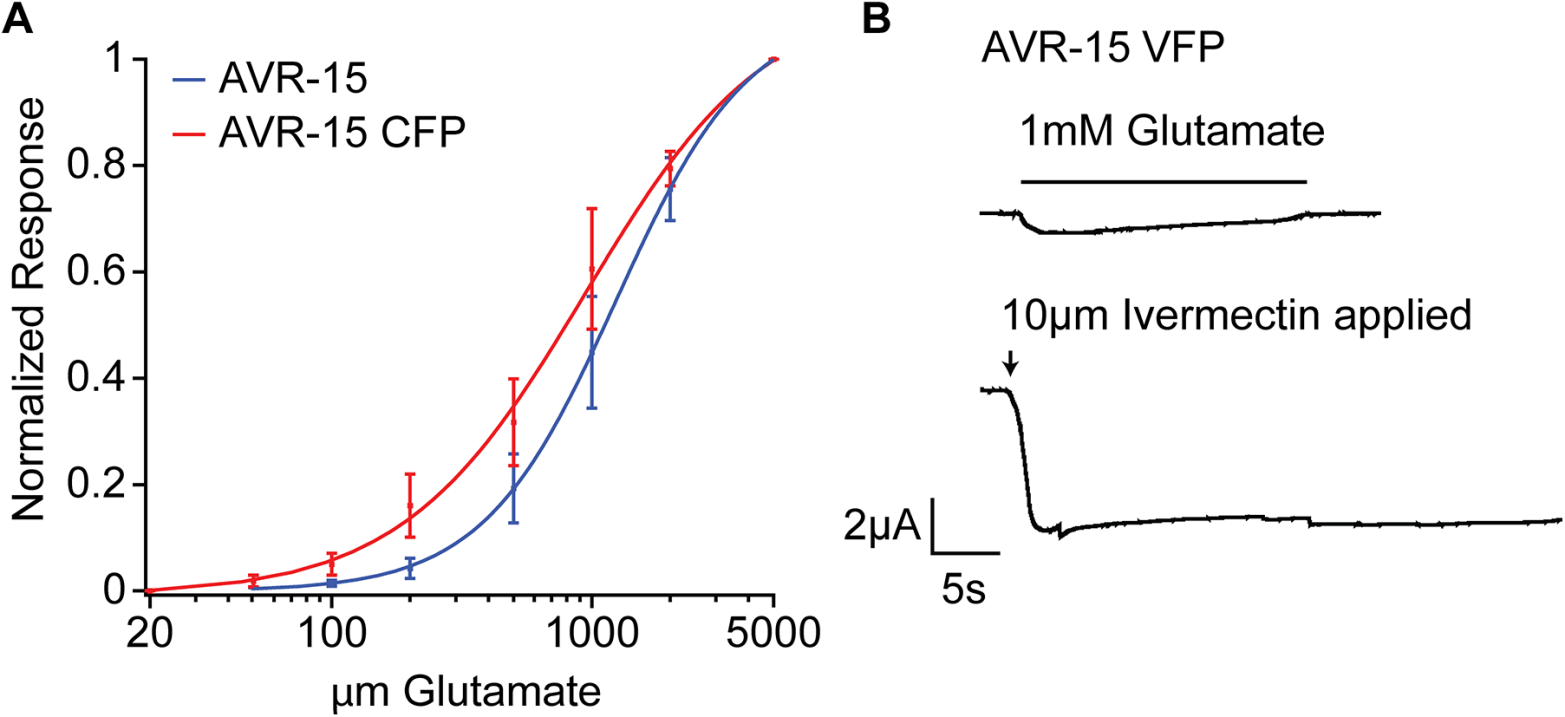
Fluorophore-tagged AVR-15 behaves electrophysiologically similar to untagged AVR-15 in Xenopus laevis oocytes. **(A)** Dose–response curve with the normalized maximal response on the y-axis plotted against the glutamate concentration on a log scale on the x-axis. The curve represents the average of three oocytes for the untagged AVR-15 and four oocytes for the fluorescently tagged AVR-15; error bars ± 1 SEM. Oocytes were clamped at –80 mV unless otherwise indicated. **(B)** An oocyte treated with 1mM glutamate and subsequently with 10μM IVM. The arrow indicates when IVM was applied (exposure to IVM was continuous from this point on) and the bar indicates when glutamate was applied. This response is similar to the untagged AVR-15 response as shown in Dent, Davis, and Avery [33]. CFP = Cerulean Fluorescent Protein; VFP = Venus Fluorescent Protein.

To confirm that the AVR-15 GluCl channel is expressed under the control of the heterologous ACC promoters, we created an AVR-15 cDNA construct with a fluorescent protein tag in the intracellular loop. When expressed in *Xenopus* oocytes, the homomeric channels formed by the tagged AVR-15 behaved electrophysiologically like the untagged AVR-15, demonstrating that the fluorescent protein tag does not affect channel assembly or overall function (Fig 4). We then made a similar YFP-tagged AVR-15 (AVR-15::YFP) construct with synthetic introns for expression in *C*. *elegans*. To increase the likelihood that the ACC promoters would faithfully reflect endogenous ACC expression, we included from one to five of the first introns of the corresponding channel subunits. To ensure portions of the ACC open reading frames from the ACC exons were not fused to the AVR-15 open reading frame, possibly interfering with AVR-15 function, we inserted an SL2 splice site between the promoter and AVR-15, making the ACC and AVR-15 gene behave as an operon. The *Pacc*::*avr-15*::YFP constructs were microinjected into worms that lack the four endogenous ivermectin-sensitive channel subunits (*avr-14*, *avr-15*, *glc-1*, *glc-3* quadruple mutant strain JD369), which display resistance to ivermectin up to 50μg/mL. The resulting transgenic strains express AVR-15 exclusively in ACC-expressing tissues, as determined by the different ACC promoters. IVM exposure activates the AVR-15 chloride channels in tissues that endogenously express the ACC channels, thus mimicking the effects of a direct ACC agonist. Because JD369 worms lack the IVM targets and thus survive exposure to IVM, a return of IVM sensitivity in the *Pacc*::*avr-15* strains indicates that the ACCs are expressed in critical tissues and could be good targets for new anthelmintics.

**Fig 5:**
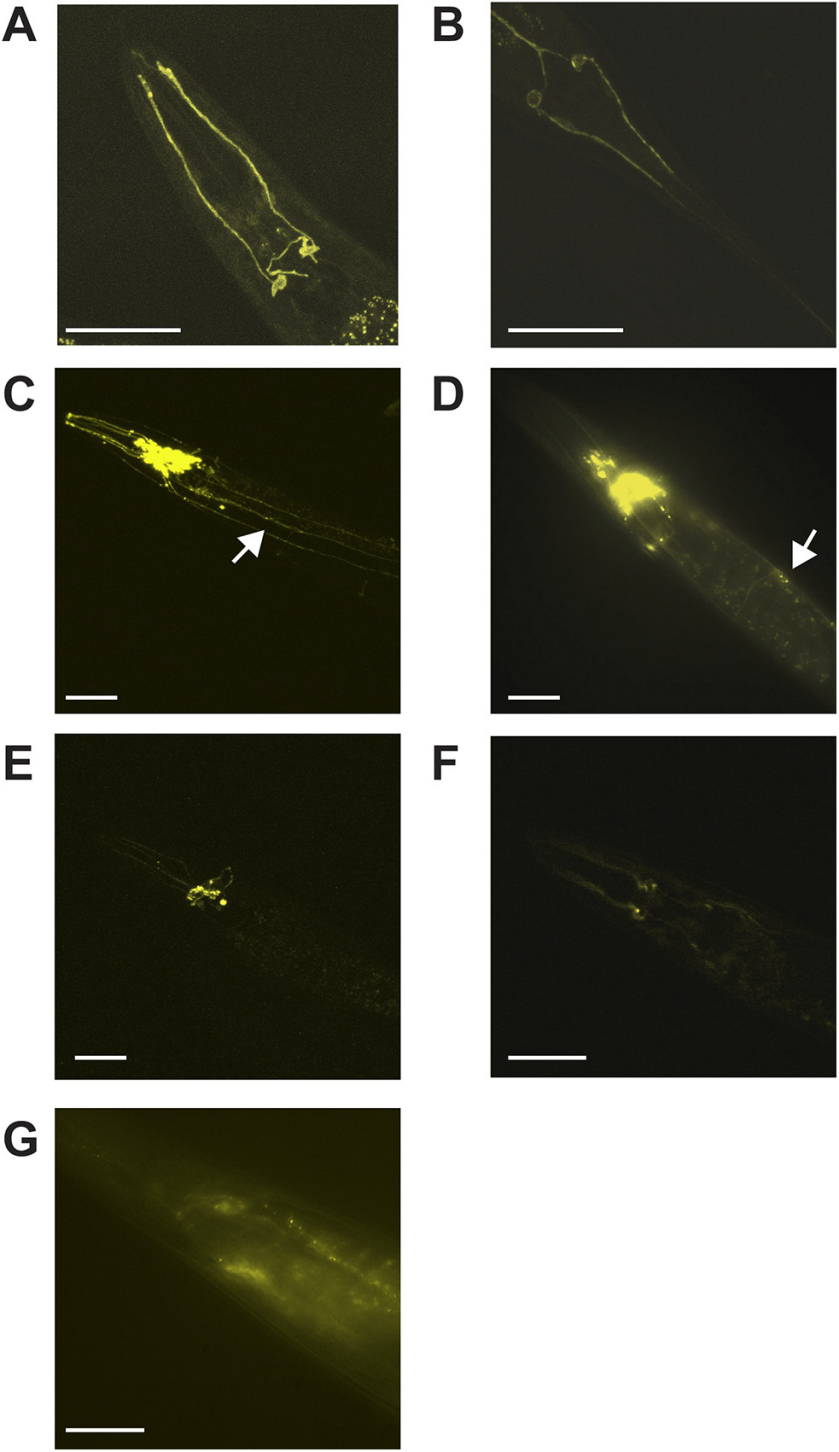
*Pacc*::AVR-15::YFP constructs are expressed in neural tissues throughout the worm. All images show representative epifluorescence of tagged YFP-tagged AVR-15 channels expressed under control of ACC promoters. **(A)** *Plgc-48*::AVR-15::YFP expression in non-essential amphid neurons in the head. **(B)** *Plgc-48*::AVR-15::YFP expression in non-essential amphid neurons in the tail. **(C)** *Plgc-47*::AVR-15::YFP expression. **(D)** *Pacc-1*::AVR-15::YFP expression. **(E)** *Pacc-2*::AVR-15::YFP expression. **(F)** *Pacc-3*::AVR-15::YFP expression. **(G)** *Plgc-49*::AVR-15::YFP expression. For all boxes, arrows indicate expression in ventral nerve cord, scale bars indicate 50μm.

We generated constructs driving AVR-15::YFP using six of the eight ACC promoters: *Pacc-1*, *Pacc-2*, *Pacc-3*, *Plgc-47*, *Plgc-49*, *and Plgc-48*, and injected them into JD369 worms. Expression of AVR-15::YFP in all strains appeared to be restricted to the nervous system. The YFP appeared to be localized to the plasma membrane, consistent with proper folding and trafficking of the AVR-15::YFP fusion protein. The LGC-48 ACC promoter (*Plgc-48*) drove expression only in two pairs of non-essential neurons, making it a suitable negative control for non-specific effects of the AVR-15::YFP transgene (Fig 5A and 5B). Notably, the other ACC transgenes appeared to be expressed in ventral cord neurons, as well as a variety of extrapharyngeal neurons (Fig 5C–5F).

**Fig 6:**
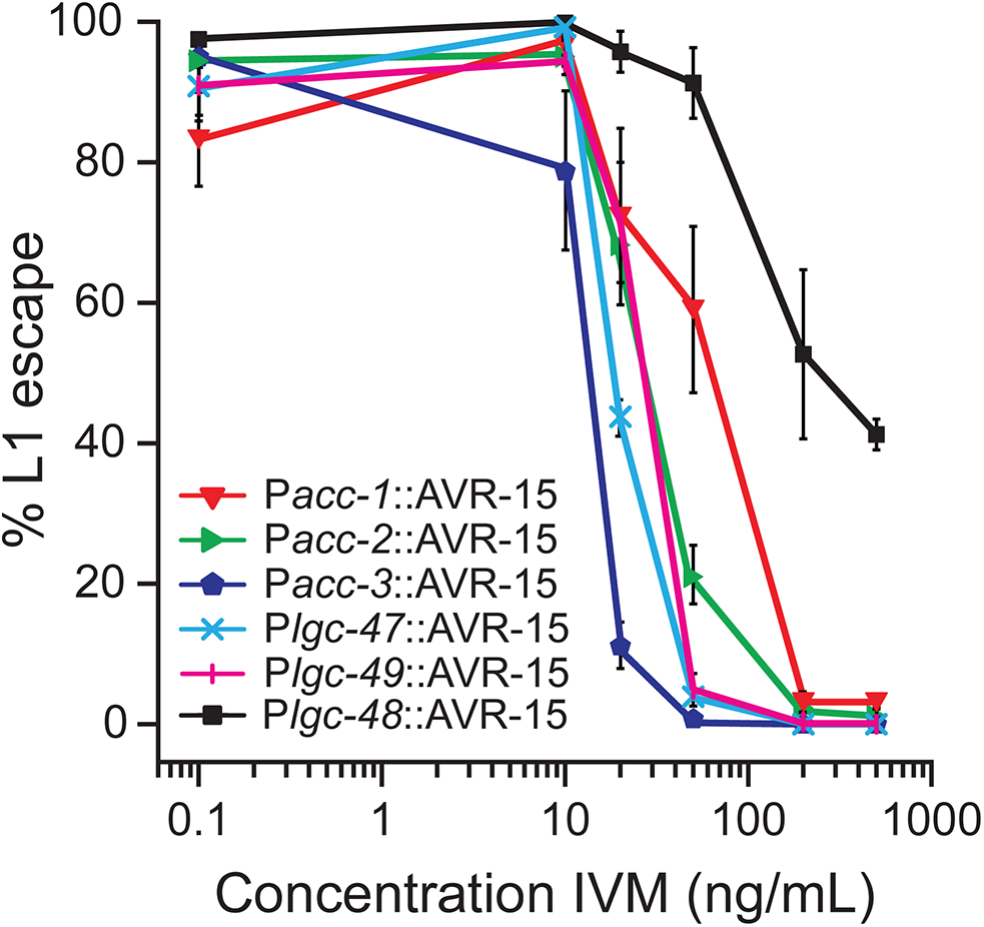
Escape from larval arrest on IVM. Worms with AVR-15::YFP expressed under control of *acc-2*, *acc-3*, *lgc-47*, and *lgc-49*-promoters fail to develop on concentrations of IVM above 200ng/mL. Percent survival was normalized to the plates with the highest total number of L1-escaped worms (either 0.1 or 10ng/mL IVM), n = 4 plates of worms per concentration, error bars ± 1 SEM.

We tested the strains on NGM plates with increasing concentrations of IVM. Eggs were placed on plates and scored for L1 escape (Fig 6). The control strains of *Plgc-48*::AVR-15::YFP had an EC_50_ = 293.0 ± 45.1ng/mL IVM while *Pacc-1*::AVR-15::YFP strains had an EC_50_ = 52.6 ± 13.5ng/mL IVM, *Pacc-2*::AVR-15::YFP strains had an EC_50_ = 28.4 ± 1.5ng/mL IVM, *Pacc-3*::AVR-15::YFP strains had an EC_50_ = 13.57 ± 0.02ng/mL IVM, *Plgc-47*::AVR-15::YFP strains had an EC_50_ = 19.3 ± 1.0ng/mL IVM, and *Plgc-49*::AVR-15::YFP strains had an EC_50_ = 25.0 ± 1.7ng/mL IVM.

While there were *Plgc*-48::AVR-15::YFP transgenic progeny that survived on IVM concentrations greater than 500ng/mL, when we exposed worms with ACC-1, ACC-2, ACC-3, LGC-47 and LGC-49 tissues expressing AVR-15 to the same concentrations of IVM, the worms died before reaching adulthood. Moreover, inactivating ACC-2-, ACC-3-, LGC-47-, and LGC-49-expressing tissues from hatching using 50ng/mL IVM resulted in very limited survival, and almost no L1 escape (S2 Fig). Worms with AVR-15 in ACC-1-expressing tissues survived at higher concentrations of IVM than the strains expressing AVR-15 in ACC-2-, ACC-3-, LGC-47-, and LGC-49-expressing tissues. We noticed, however, that at sub-lethal concentrations of IVM, the worms with AVR-15 in ACC-1-expressing tissues were paralyzed, a phenotype consistent with ACC-1 expression in the ventral nerve cord.

**Fig 7:**
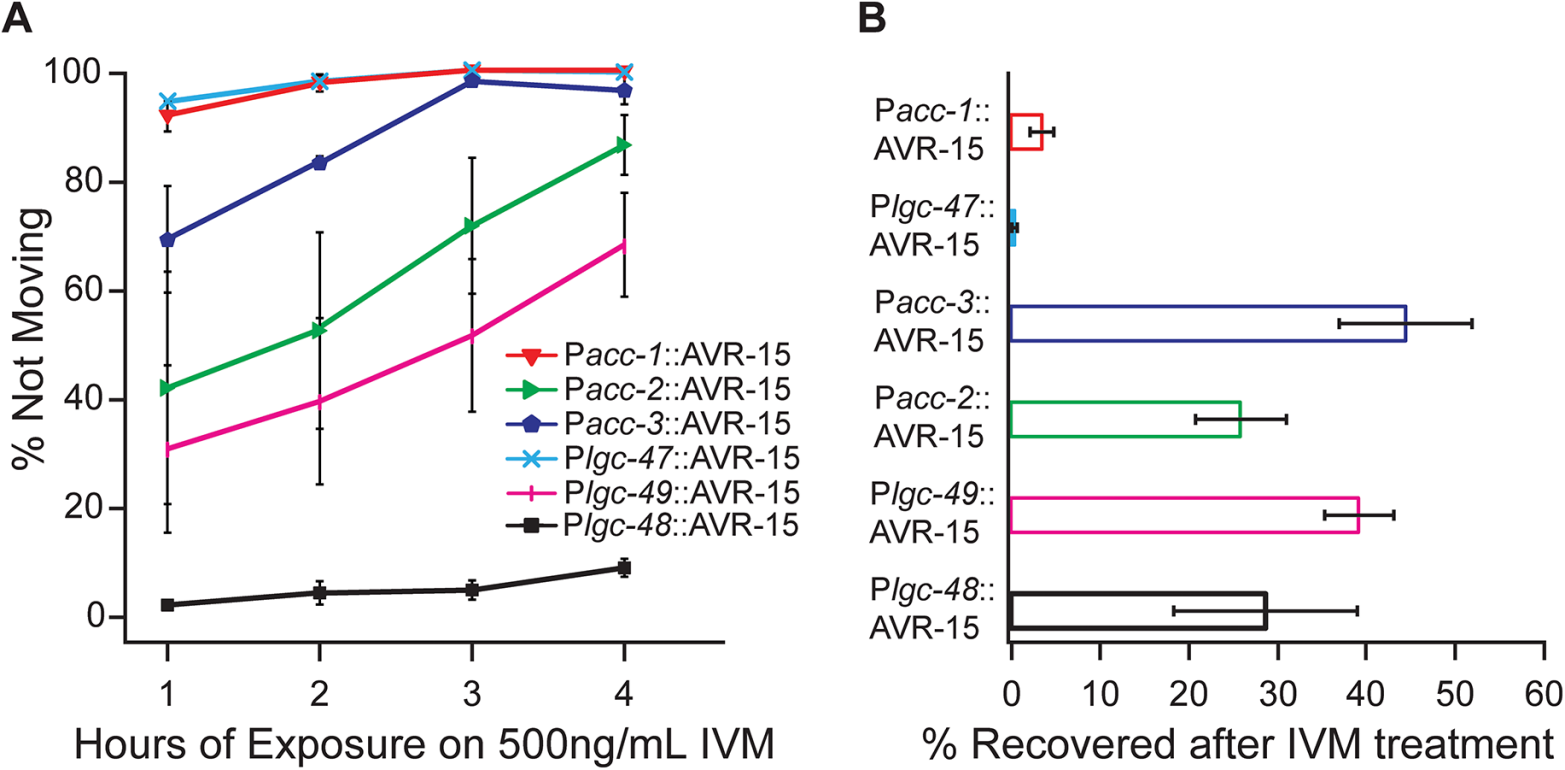
Adult paralysis on IVM. **(A)** Well-fed young adults were placed on 500ng/mL IVM and were monitored every hour up to four hours for their ability to move in response to a plate tap or stimulation with a worm pick (harsh touch). All strains, except *Plgc*-48::AVR-15 containing strains, showed paralysis after four hours on 500ng/mL IVM. n = 4 plates per genotype. **(B)** After 4h of 500ng/mL IVM exposure, paralyzed worms were allowed to recover overnight and worms that moved in response to a plate tap or stimulation with a worm pick (harsh touch) were scored as recovered. n = 4 plates per genotype, error bars ± 1 SEM.

Because drugs affecting nematode movement are used to help clear parasitic infections, we quantified acute effects of IVM on adult movement. After 1h on 500ng/mL IVM plates, 92 ± 3% of *Pacc-1*::AVR-15::YFP adults, 42 ± 21% of *Pacc-2*::AVR-15::YFP adults, 69 ±10% of *Pacc3*::AVR-15::YFP adults, 95 ± 1% of *Plgc-47*::AVR-15::YFP adults, and 31 ± 15% of *Plgc-49*::AVR-15::YFP adults were paralyzed, compared to the control strain of *Plgc-48*::AVR-15::YFP worms which showed 2 ± 1% of adult worms paralysis (Fig 7A). These percentages increased with time, and by four hours of exposure to 500ng/mL IVM, most worms from all the strains, except for the *Plcg-48*::AVR-15::YFP strains, had stopped moving, with 100% of *Pacc-1*::AVR-15::YFP adults, 87 ± 6% of *Pacc-2*::AVR-15::YFP adults, 97 ± 3% of *Pacc-3*::AVR-15::YFP adults, 100% of *Plgc-47*::AVR-15::YFP adults, 68 ± 10% of *Plgc-49*::AVR-15::YFP adults and 9 ± 2% of *Plgc-48*:: AVR-15::YFP adults paralyzed. To see if pACC::AVR-15::YFP-expressing strains recover from IVM treatment, after 4h on IVM the worms were transferred back onto seeded NGM plates, left overnight, and scored for recovery of movement. The severely paralyzed *Plgc-47*::AVR-15::YFP and *Pacc-1*::AVR-15::YFP adults did not recover (0.5 ± 0.3% and 3.5 ± 1.3% recovery respectively). The other strains had slightly higher recovery rates with 25.9 ± 5.1% of *Pacc-2*::AVR-15::YFP adults, 44.5 ± 7.5% of *Pacc-3*::AVR-15::YFP adults, 39.3 ± 4.0% of *Plgc-49*::AVR-15::YFP adults and 28.7 ± 10.4% of *Plgc-48*:: AVR-15::YFP adults recovering the ability to move after being left to recover overnight from 4h IVM exposure (Fig 7B).

**Fig 8:**
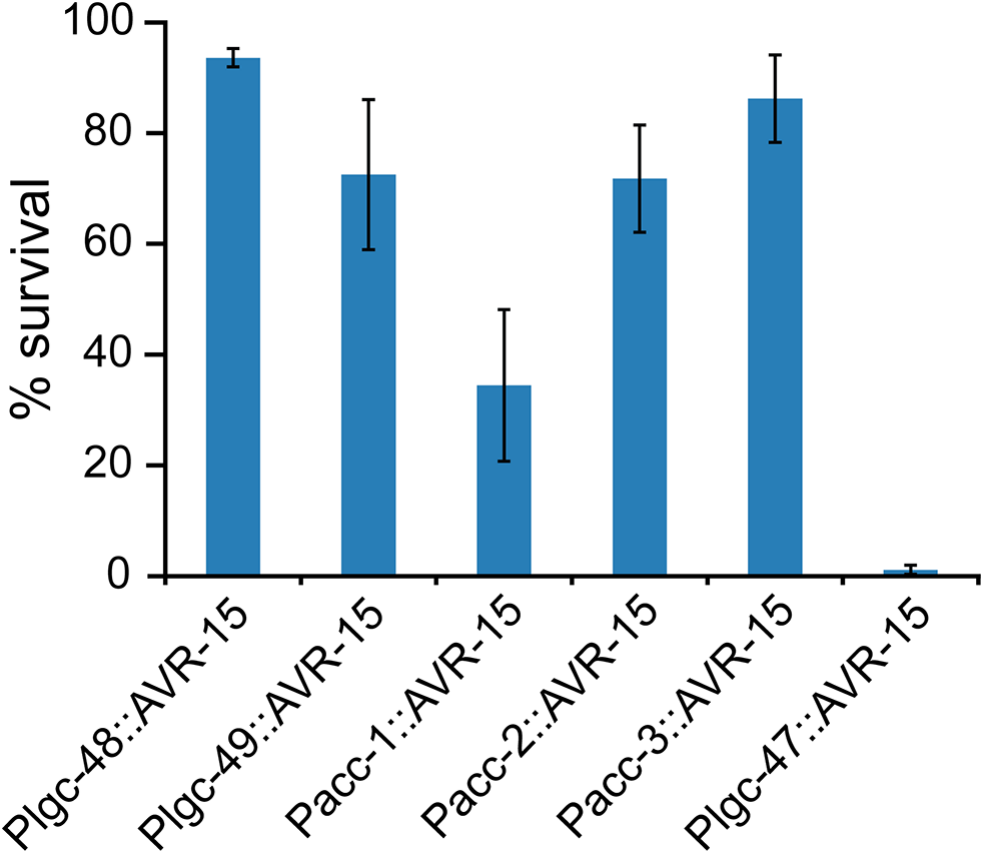
Adulticidal IVM activity. Well-fed young adults were placed on 500ng/mL IVM and were checked for live worms every day up to five days. Survival was assessed as described in Wilkinson et al. [38]. Graph shows adult survival after 5 days on 500ng/mL IVM. Strains with *Plgc*-47::AVR-15 were unable to survive to day five, while all other strains, except *Plgc*-48::AVR-15 containing strains, showed reduced survival, n = 3 plates per genotype, error bars ± 1 SEM.

Since successful treatment of nematode parasitic infections requires killing or clearing animals of various developmental stages, we were also interested in whether an ACC agonist would be likely to have adulticidal activity. To test this, we placed IVM-naïve adults of the *Pacc*::AVR-15::YFP- expressing strains on IVM. All of the strains placed on the IVM plates had adults that survived past day five on 500ng/mL IVM, except for the *Plgc-47*::AVR-15::YFP strains, which had extremely limited survival (1.1 ± 0.8%) after five days on IVM plates (Fig 8). *Plgc-48*::AVR-15::YFP adults were largely unaffected by 500ng/mL IVM with an average survival of 93 ± 1.6% after 5 days. *Pacc-2*:: and *Pacc-3*::AVR-15::YFP adults struggled on IVM; however, most of the adults were still alive, exhibiting survival rates of 71.8 ± 9.6% and 86.2 ± 7.9%, respectively. Similarly, 72.5 ± 13.7% of *Plgc-49*::AVR-15::YFP adult worms survived 5 days of 500ng/mL IVM exposure. *Pacc-1*::AVR-15::YFP adult worms also had reduced survival (34.5 ± 13.7%) after five-day exposure to 500ng/mL IVM, and they were severely paralyzed.

## Discussion

We developed a novel validation strategy for potential ion channel targets of anthelmintic drugs. We used the IVM-gated AVR-15 channel to validate five members of the *acc* gene family in *C*. *elegans* as appropriate targets for the development of new anthelmintics and believe this approach can be used to validate ion channel targets in any species that is amenable to transgenic manipulation. Our assay is an enabling technology that provides direct validation to support the development of novel ion channel-based drug screens.

### Drug Target Validation Strategies

Target validation prior to undertaking high-throughput drug screens ensures that hit compounds will have the desired *in vivo* effect. Genetic approaches to target validation have demonstrated their utility in systems as diverse as cholesterol metabolism [44] and HIV [45]. The underlying principle is that loss-of-function alleles of a gene (e.g. a knockout) mimic the effects of a small molecule antagonist and hypermorphic or pathway-activating mutations mimic agonists.

We were interested in developing a genetic strategy in *C*. *elegans* to validate ligand-gated ion channels as suitable targets for novel anthelmintic agonists or antagonists. Most successful anthelmintics are channel agonists, and the genetic evidence suggests that antagonists of ivermectin receptors and monepantel receptors would not be efficacious anthelmintics. Ivermectin is an agonist/activator of GluCls, which are themselves, non-essential targets; *avr-14*, *avr-15*, *glc-1*, *glc-3* quadruple mutants are insensitive to ivermectin, are viable and only display subtle behavioral phenotypes [33,46–53]. Similarly, the monepantel-resistant *C*. *elegans acr-23* knockout strains have no obvious phenotype [54], while monepantel-induced over-activation of ACR-23 is an effective anti-parasitic strategy. In contrast, levamisole-resistant mutants, primarily knockout alleles of the levamisole-sensitive nicotinic-type acetylcholine receptor subunits, have a non-lethal uncoordinated locomotion phenotype [55] consistent with their role mediating cholinergic neurotransmission at the neuromuscular junction. The knockout phenotype indicates that a levamisole receptor antagonist could be an effective anthelmintic, a prediction that was confirmed with the discovery of the anthelmintic derquantel [56] and the fact that paraherquamide and its analog 2-deoxy-paraherquamide are also competitive cholinergic antagonists [57]. Presumably, for neurotransmitter receptor ion channels the greater toxic potential of agonists is because an antagonist interferes with a subset of synapses, degrading information processing, whereas an agonist renders the entire excitable cell non-functional. However, if the synaptic transmission blocked by an antagonist is sufficiently widespread and/or critical to vital behaviors, as in the case of levamisole/derquantel receptors, an antagonist will also be an effective anthelmintic. Hence, we predict that the neurotransmitter receptors for which an antagonist will be an effective anthelmintic will always be a subset of the receptors for which an agonist will be effective. Consistent with that prediction, we showed that knockouts of individual ACCs had only modest effects on viability in *C*. *elegans* and therefore an ACC antagonist is less likely to be an effective anti-parasitic drug. It is possible, however, that unlike the GluCls, the ACCs have redundant essential functions and that a drug that inactivated many ACC channels simultaneously would have deleterious effects on the worm.

Hypermorphic or gain-of-function alleles can, in principle, reveal the effect of an agonist, however the technology for generating hypermorphs, specifically the ability to predict what mutations will be hypermorphic, is often inaccessible. In addition, a mutation present from birth raises issues of adaptation or developmental effects that can affect the interpretation of phenotypes, leading to the development of conditional mutations. We have ectopically expressed the IVM-activated chloride channel AVR-15 under control of ACC promoters and treated the worms with IVM, allowing precise temporal and spatial control of tissue inactivation. This approach was used to infer the effects of an ACC-agonist on a model nematode, and could be used to validate other ion channels as suitable targets of agonist drugs.

### The ACCs are Validated Targets of Agonists

Our results indicate that that the ACCs are promising targets for anthelmintic discovery. Constitutive activation of a chloride channel in ACC-2-, ACC-3-, LGC-47- and LGC-49- expressing tissues was lethal or resulted in arrested or severely delayed development of *C*. *elegans* larvae. These ACC targets therefore have potential as larvicides for treatment of filariases and microfilariae. Treatment of infections with gastrointestinal nematodes requires expulsion of the adults from the gastrointestinal tract, which can be achieved by affecting the motility of the worms. We found that ivermectin-induced inhibition of tissues expressing ACC-1, or LGC-47, resulted in rapid and severe paralysis. Based on the AVR-15::YFP expression patterns, both of these subunits appear to be expressed in neurons innervating the ventral nerve cord, a key circuit in coordination of locomotion. Thus, we predict agonists of ACC-1 and/or LGC-47 would be efficacious against infections with gastrointestinal nematodes. It is also important to note that we validated individual ACC subunits. A drug that acted on many members of the structurally similar ACC family would likely exhibit greater efficacy and would be less susceptible to the evolution of receptor-mediated drug resistance, although ultimately, the efficacy of an ACC agonist would depend on the physiological roles of the tissues in which the ACCs are expressed.

Interestingly, the of potency of the AVR-15 expressing strains varied significantly, even though the potency might be expected to reflect the affinity of ivermectin for AVR-15, which should be the same in all strains. Instead, the variation in potency, as measured by whole organism phenotypes, may reflect the pharmacokinetics of ivermectin–internal tissues may experience different ivermectin concentrations at a given extra-cuticular ivermectin exposure. Similarly, the recovery from ivermectin treatment (Fig 7B) may be an indication of pharmacokinetics; strains recovering from ivermectin treatment may express AVR-15 in tissues that experience lower ivermectin concentrations or higher rates of detoxification.

Adulticidal compounds that are not potent larvicides are also of interest for the control of filariases as part of mass drug administration programs. Ivermectin, which is currently the cornerstone of mass drug administration programs for the control of onchocerciasis, only kills microfilaria (larvae). A compound that would also kill adult parasites, which can live up to a decade, would obviate the need for repeat treatments to control symptoms caused by microfilaria. Our results indicate that an LGC-47 agonist would be the most potent adulticide, albeit after prolonged drug exposure. However, the efficacy of inhibition of larval growth was still much greater than the adulticidal activity, indicating that ACC agonists would likely be more effective larvicides than adulticdes.

### *C*. *elegans* as a Model for Validation of Potential Anti-Parasitic Drug Targets

The choice of *C*. *elegans* as a model for parasitic nematodes is subject to several caveats. First, the target must be widely conserved among nematodes. The conservation of homologous genes among *C*. *elegans* and parasitic nematodes varies across the diverse phylum, ranging from 35% -70% [58]. Importantly, we show that the ACCs are highly conserved, even in the most distantly related parasitic species (e.g. *Trichinella spiralis*). Second, the anatomy among nematodes should be conserved. Neural anatomy of parasites has not been sufficiently well-characterized to make a clear determination in this regard, although, *Haemonchus contortus* amphid neurons have been reconstructed by electron microscopy and appear to have close correspondence with *C*. *elegans* amphid neurons [59]. Similarly, the ventral cord anatomy of *Ascaris suum* has been characterized electrophysiologically and is analogous to that of *C*. *elegans* [60–62]. Finally, target expression must be broadly conserved across nematode species. Using GluCls as an example, while GluCl ortholog expression patterns are not perfectly conserved between *Haemonchus contortus* and *C*. *elegans*, their expression in essential tissues and overall functions appear to be conserved [63,64]. Moreover, *C*. *elegans* and *H*. *contortus* both show inhibition of pharyngeal pumping at comparable, physiologically-relevant, ivermectin concentrations in vitro [65–67], although *C*. *elegans* appears more sensitive to the locomotor effects [65]. The size of the GluCl family and the conservation of specific subunit orthologs, however, has also been shown to vary across nematode species [22,47]. Changes in the size and/or expression patterns of the GluCl gene family may explain the differences in sensitivity of *C*. *elegans* and the filarial species to the effects of IVM on locomotion in microfilaria and adults as well as effects on larval development in vitro [68–72]. Although IVM kills adult *C*. *elegans*, but not adult filaria, it does so preventing pharyngeal pumping resulting in starvation [33], a mechanism that predictably would not be relevant to subcuticular parasites that may not require the pharynx to absorb nutrients in vivo, even if GluCl expression in the pharynx is conserved. If the sterilizing effect of IVM in adult female filaria in vivo is primarily a result of inhibition of male mating [69, 72], then the paralysis of *C*. *elegans* males by IVM may reflect the conservation of GluCl expression in the male mating/locomotor circuit. Finally, to the extent that an anthelmintic exerts its antiparasitic effect by altering the interaction of the parasite with its host or with a symbiont, those mechanisms will not be reflected in the *C*. *elegans* model [68,71,73].

The use of the AVR-15 channel to mimic activation of an endogenous chloride channel also has associated caveats. For instance, the transgenic strains we used contain multiple copies of the transgene (extrachromosomal arrays), and therefore the transgene is typically overexpressed, possibly resulting in an overestimate of the severity of the phenotype when compared to an agonist of the endogenous channel. However, *C*. *elegans* neurons are thought to be nearly isopotential, and have high enough input resistance, that a single ion channel is sufficient to change the membrane potential of a neuron [74]. Thus, even a modestly-expressed ion channel target, when activated by a drug with comparable effectiveness to IVM, is likely to clamp the membrane to an unexcitable potential and/or act as a potent shunt of excitation. Similarly, endogenous channels may be synaptically targeted, resulting in localized effects, whereas AVR-15::YFP appears to be uniformly distributed in the membrane. However, again, the putative isopotentiality of neurons likely minimizes synapse-specific effects. Finally, leaky expression of AVR-15 from our promoter fusions may lead to over-estimation of the sensitivity of the transgenic strains to activation of endogenous channels. The *Plgc*-48::*avr-15*::YFP strain allowed us to estimate an upper limit on the lethality attributable to general, low-level, leaky expression of the AVR-15::YFP construct, and we propose that the added lethality seen in our other *Pacc*::*avr-15*::YFP strains is exclusively due to inactivation of those ACC-expressing tissues. Overexpression of the transgenes, and the associated potentially negative consequences, could be addressed by creating strains with targeted, single-copy insertions of the transgenes, especially now that the technology for such fine-tuned genetic manipulation has become available in many different species [75–80].

In order to use IVM in our assay, we had to perform all the experiments in an IVM-resistant quadruple GluCl mutant background (strain JD369). As previously mentioned, JD369 worms are viable, fertile and only have very subtle phenotypes. Despite this fact, the IVM resistant background necessary for these experiments may complicate the translation of this technique for validation of chloride channel targets in other species with endogenous IVM targets. While these factors will tend to result in less conservative estimates of target sensitivity to agonists, we are reassured by the similarity of the ACC neuronal expression patterns to endogenous anthelmintic targets such as AVR-14, another pLGIC subunit targeted by IVM [46].

There remain many uncharacterized pLGICs in nematodes and, along with the subunits that have been characterized but are not currently targets of anthelmintics, it is clear that many novel nematode pLGIC subunits could be validated as suitable drug targets. In principle the technique that we presented here to validate the ACCs could be used to validate agonists of other chloride-selective ion channels in any organism or strain amenable to creation of transgenics and that is not otherwise ivermectin sensitive. The validation strategy we describe expands opportunities to employ target-selective screening of ion channels.

## Supporting Information

S1 Fig Deletions in *acc* genes. Depiction of the deleted genomic regions in the strains vc1757 (*Δacc-2*), vc40013 (Δ*lgc-49)*, rb2187 (Δ*lgc-47)*, and tm3268 (*Δacc-1*).
(DOCX)

S2 Fig Growth of strains on 50ng/mL IVM on day three. Worms with AVR-15::YFP expressed under control of *acc-2*, *acc-3*, *lgc-47*, and *lgc-49-*promoters fail to develop on 50ng/mL IVM. Worms with AVR-15::YFP expressed under control of *lgc-48*-promoter develop to adulthood, while worms with AVR-15::YFP expressed exclusively in ACC-1-expressing tissues exhibit delayed growth on 50ng/mL IVM, but do reach adulthood.
(DOCX)

S1 Table ACC Orthologs. Table listing all genes used in constructing the ACC phylogeny (Fig 1). Included for each gene is the corresponding GI accession number, GenBank accession number, the original name assigned to that gene, the organism in which that gene was found, and the new ACC name we have assigned to the gene.
(DOCX)

S2 Table Cloning Primers. Table describing all primers used to clone all the acc promoters used in the acc::AVR-15 constructs. Gene-specific sequence in lowerase, vector-specific sequence in uppercase.
(DOCX)

S3 Table Screening Primers. Primers used to screen for deletions in strains vc1757 (*Δacc-2*), vc40013 (Δ*lgc-49)*, rb2187 (Δ*lgc-47)*, and tm3268 (*Δacc-1*).
(DOCX)

S4 Table Worm strains used. Table listing all the worm strains used, including a list of the alleles each strain is carrying.
(DOCX)

## Abbreviations

ACCs: acetylcholine-gated chloride channels
pLGIC: pentameric ligand-gated ion channel
FP: fluorescent protein
GFP: green fluorescent protein
YFP: yellow fluorescent protein
IVM: ivermectin
nAChR: nicotinic acetylcholine receptor
GluCl: glutamate-gated chloride channels
L1: larval stage 1
EC_50_: half maximal effective concentration

## References

[1] World Health Organization. WHO | Soil-transmitted helminth infections. In: Fact sheet N°366 [Internet]. Fact sheet. World Health Organization; 2014. Available: http://www.who.int/mediacentre/factsheets/fs366/en/. Accessed 2014 Jun 13.

[2] Atkinson HJ, Lilley CJ, Urwin PE. Strategies for transgenic nematode control in developed and developing world crops. Curr Opin Biotechnol. 2012;23: 251–6. 10.1016/j.copbio.2011.09.004 21996368

[3] Sutherland IA, Leathwick DM. Anthelmintic resistance in nematode parasites of cattle: a global issue? Trends Parasitol. 2011;27: 176–81. 10.1016/j.pt.2010.11.008 21168366

[4] Osei-Atweneboana MY, Eng JKL, Boakye DA, Gyapong JO, Prichard RK. Prevalence and intensity of Onchocerca volvulus infection and efficacy of ivermectin in endemic communities in Ghana: a two-phase epidemiological study. Lancet. 2007;369: 2021–9. 10.1016/S0140-6736(07)60942-8 17574093

[5] Beech RN, Skuce P, Bartley DJ, Martin RJ, Prichard RK, Gilleard JS. Anthelmintic resistance: markers for resistance, or susceptibility? Parasitology. Cambridge University Press; 2011;138: 160–74. 10.1017/S0031182010001198 20825689 PMC3064440

[6] Sangster NC. Managing parasiticide resistance. Vet Parasitol. 2001;98: 89–109. Available: http://www.ncbi.nlm.nih.gov/pubmed/11516581 11516581 10.1016/s0304-4017(01)00425-3

[7] Kaplan RM. Drug resistance in nematodes of veterinary importance: a status report. Trends Parasitol. 2004;20: 477–81. 10.1016/j.pt.2004.08.001 15363441

[8] Hudson A, Nwaka S. The Concept Paper on the Helminth Drug Initiative. Onchocerciasis/lymphatic filariasis and schistosomiasis: opportunities and challenges for the discovery of new drugs/diagnostics. Expert Opin Drug Discov. 2007;2: S3–7. 10.1517/17460441.2.S1.S3 23489030

[9] Nwaka S, Hudson A. Innovative lead discovery strategies for tropical diseases. Nat Rev Drug Discov. Nature Publishing Group; 2006;5: 941–55. 10.1038/nrd2144 17080030

[10] Nwaka S, Besson D, Ramirez B, Maes L, Matheeussen A, Bickle Q, et al Integrated Dataset of Screening Hits against Multiple Neglected Disease Pathogens. Keiser J, editor. PLoS Negl Trop Dis. 2011;5: e1412 10.1371/journal.pntd.0001412 22247786 PMC3243694

[11] Laing R, Kikuchi T, Martinelli A, Tsai IJ, Beech RN, Redman E, et al The genome and transcriptome of Haemonchus contortus, a key model parasite for drug and vaccine discovery. Genome Biol. BioMed Central Ltd; 2013;14: R88 10.1186/gb-2013-14-8-r88 23985316 PMC4054779

[12] Mitreva M, Jasmer DP, Zarlenga DS, Wang Z, Abubucker S, Martin J, et al The draft genome of the parasitic nematode Trichinella spiralis. Nat Genet. Nature Publishing Group; 2011;43: 228–35. 10.1038/ng.769 PMC3057868 21336279

[13] Jex AR, Nejsum P, Schwarz EM, Hu L, Young ND, Hall RS, et al Genome and transcriptome of the porcine whipworm Trichuris suis. Nat Genet. 2014;46: 701–6. 10.1038/ng.3012 24929829 PMC4105696

[14] Schwarz EM, Korhonen PK, Campbell BE, Young ND, Jex AR, Jabbar A, et al The genome and developmental transcriptome of the strongylid nematode Haemonchus contortus. Genome Biol. 2013;14: R89 10.1186/gb-2013-14-8-r89 23985341 PMC4053716

[15] Godel C, Kumar S, Koutsovoulos G, Ludin P, Nilsson D, Comandatore F, et al The genome of the heartworm, Dirofilaria immitis, reveals drug and vaccine targets. The FASEB Journal. 2012 pp. 4650–4661. 10.1096/fj.12-205096 22889830 PMC3475251

[16] Jex AR, Liu S, Li B, Young ND, Hall RS, Li Y, et al Ascaris suum draft genome. Nature. Nature Publishing Group; 2011;479: 529–33. 10.1038/nature10553 22031327

[17] Ghedin E, Wang S, Spiro D, Caler E, Zhao Q, Crabtree J, et al Draft genome of the filarial nematode parasite Brugia malayi. Science. 2007;317: 1756–1760. 17885136 10.1126/science.1145406 PMC2613796

[18] Opperman CH, Bird DM, Williamson VM, Rokhsar DS, Burke M, Cohn J, et al Sequence and genetic map of Meloidogyne hapla: A compact nematode genome for plant parasitism. Proc Natl Acad Sci U S A. 2008;105: 14802–14807. 10.1073/pnas.0805946105 18809916 PMC2547418

[19] Abad P, Gouzy J, Aury J-M, Castagnone-Sereno P, Danchin EGJ, Deleury E, et al Genome sequence of the metazoan plant-parasitic nematode Meloidogyne incognita. Nat Biotechnol. Nature Publishing Group; 2008;26: 909–915. 10.1038/nbt.1482 18660804

[20] Desjardins C, Cerqueira GC, Goldberg JM, Dunning Hotopp JC, Haas BJ, Zucker J, et al Genomics of Loa loa, a Wolbachia-free filarial parasite of humans. Nat Genet. 2013;45: 495–500. 10.1038/ng.2585 23525074 PMC4238225

[21] Foth BJ, Tsai IJ, Reid AJ, Bancroft AJ, Nichol S, Tracey A, et al Whipworm genome and dual-species transcriptome analyses provide molecular insights into an intimate host-parasite interaction. Nat Genet. Nature Publishing Group, a division of Macmillan Publishers Limited. All Rights Reserved.; 2014;46: 693–700. 10.1038/ng.3010 24929830 PMC5012510

[22] Williamson SM, Walsh TK, Wolstenholme AJ. The cys-loop ligand-gated ion channel gene family of Brugia malayi and Trichinella spiralis: a comparison with Caenorhabditis elegans. Invert Neurosci. 2007;7: 219–26. 10.1007/s10158-007-0056-0 17952476

[23] Abuin A, Holt KH, Platt KA, Sands AT, Zambrowicz BP. Full-speed mammalian genetics: in vivo target validation in the drug discovery process. Trends Biotechnol. 2002;20: 36–42. 10.1016/S0167-7799(01)01843-1 11742676

[24] Hartwell LH. Integrating Genetic Approaches into the Discovery of Anticancer Drugs. Science (80-). 1997;278: 1064–1068. 10.1126/science.278.5340.1064 9353181

[25] Miesel L, Greene J, Black TA. Genetic strategies for antibacterial drug discovery. Nat Rev Genet. 2003;4: 442–56. 10.1038/nrg1086 12776214

[26] Plenge RM, Scolnick EM, Altshuler D. Validating therapeutic targets through human genetics. Nat Rev Drug Discov. Nature Publishing Group, a division of Macmillan Publishers Limited. All Rights Reserved.; 2013;12: 581–94. 10.1038/nrd4051 23868113

[27] Raymond-Delpech V, Matsuda K, Sattelle BM, Rauh JJ, Sattelle DB. Ion channels: molecular targets of neuroactive insecticides. Invert Neurosci. Springer Berlin / Heidelberg; 2005;5: 119–33. 10.1007/s10158-005-0004-9 16172884

[28] Dent JA. Evidence for a diverse Cys-loop ligand-gated ion channel superfamily in early bilateria. J Mol Evol. Springer New York; 2006;62: 523–35. 10.1007/s00239-005-0018-2 16586016

[29] Putrenko I, Zakikhani M, Dent JA. A family of acetylcholine-gated chloride channel subunits in Caenorhabditis elegans. J Biol Chem. 2005;280: 6392–8. 10.1074/jbc.M412644200 15579462

[30] Frazier SJ, Cohen BN, Lester HA. An engineered glutamate-gated chloride (GluCl) channel for sensitive, consistent neuronal silencing by ivermectin. J Biol Chem. 2013;288: 21029–42. 10.1074/jbc.M112.423921 23720773 PMC3774370

[31] Guindon S, Dufayard J-F, Lefort V, Anisimova M, Hordijk W, Gascuel O. New algorithms and methods to estimate maximum-likelihood phylogenies: assessing the performance of PhyML 3.0. Syst Biol. 2010;59: 307–21. 10.1093/sysbio/syq010 20525638

[32] Beech RN, Wolstenholme AJ, Neveu C, Dent J. Nematode parasite genes: what’s in a name? Trends Parasitol. Elsevier Ltd; 2010;26: 334–40. 10.1016/j.pt.2010.04.003 20478743

[33] Dent JA, Davis MW, Avery L. avr-15 encodes a chloride channel subunit that mediates inhibitory glutamatergic neurotransmission and ivermectin sensitivity in Caenorhabditis elegans. EMBO J. 1997;16: 5867–79. 10.1093/emboj/16.19.5867 9312045 PMC1170218

[34] Mello CC, Kramer JM, Stinchcomb D, Ambros V. Efficient gene transfer in C.elegans: extrachromosomal maintenance and integration of transforming sequences. EMBO J. 1991;10: 3959–70. Available: http://www.pubmedcentral.nih.gov/articlerender.fcgi?artid=453137&tool=pmcentrez&rendertype=abstract 1935914 10.1002/j.1460-2075.1991.tb04966.x PMC453137

[35] Mello C, Fire A. DNA transformation. Methods Cell Biol. 1995;48: 451–82. Available: http://www.ncbi.nlm.nih.gov/pubmed/8531738 8531738

[36] Brenner S. The genetics of Caenorhabditis elegans. Genetics. 1974;77: 71–94. Available: http://www.pubmedcentral.nih.gov/articlerender.fcgi?artid=1213120&tool=pmcentrez&rendertype=abstract 4366476 10.1093/genetics/77.1.71 PMC1213120

[37] Wood WB, ed. The Nematode Caenorhabditis elegans New York: Cold Spring Harbor Laboratory Press; 1988.

[38] Wilkinson DS, Taylor RC, Dillin A. Analysis of aging in Caenorhabditis elegans. Methods Cell Biol. 2012;107: 353–81. 10.1016/B978-0-12-394620-1.00012-6 22226530

[39] Blaxter M. Nematodes: the worm and its relatives. PLoS Biol. 2011;9: e1001050 10.1371/journal.pbio.1001050 21526226 PMC3079589

[40] Köhler P. The biochemical basis of anthelmintic action and resistance. Int J Parasitol. 2001;31: 336–345. 10.1016/S0020-7519(01)00131-X 11400692

[41] Martin RJ. Modes of action of anthelmintic drugs. Vet J. 1997;154: 11–34. 10.1016/S1090-0233(05)80005-X 9265850

[42] Arena JP, Liu KK, Paress PS, Frazier EG, Cully DF, Mrozik H, et al The Mechanism of Action of Avermectins in Caenorhabditis Elegans: Correlation between Activation of Glutamate-Sensitive Chloride Current, Membrane Binding, and Biological Activity. J Parasitol. The American Society of Parasitologists; 1995;81: 286–294. 10.2307/3283936 7707209

[43] Cully DF, Vassilatis DK, Liu KK, Paress PS, Van der Ploeg LH, Schaeffer JM, et al Cloning of an avermectin-sensitive glutamate-gated chloride channel from Caenorhabditis elegans. Nature. 1994;371: 707–11. 10.1038/371707a0 7935817

[44] Tobert JA. Lovastatin and beyond: the history of the HMG-CoA reductase inhibitors. Nat Rev Drug Discov. 2003;2: 517–26. 10.1038/nrd1112 12815379

[45] Hariharan M, Scaria V, Pillai B, Brahmachari SK. Targets for human encoded microRNAs in HIV genes. Biochem Biophys Res Commun. 2005;337: 1214–8. 10.1016/j.bbrc.2005.09.183 16236258

[46] Dent JA. The genetics of ivermectin resistance in Caenorhabditis elegans. Proc Natl Acad Sci. 2000;97: 2674–2679. 10.1073/pnas.97.6.2674 10716995 PMC15988

[47] Yates DM, Portillo V, Wolstenholme AJ. The avermectin receptors of Haemonchus contortus and Caenorhabditis elegans. Int J Parasitol. 2003;33: 1183–1193. 10.1016/S0020-7519(03)00172-3 13678634

[48] Hukema RK, Rademakers S, Jansen G. Gustatory plasticity in C. elegans involves integration of negative cues and NaCl taste mediated by serotonin, dopamine, and glutamate. Learn Mem. 2008;15: 829–36. 10.1101/lm.994408 18984564

[49] Ohnishi N, Kuhara A, Nakamura F, Okochi Y, Mori I. Bidirectional regulation of thermotaxis by glutamate transmissions in Caenorhabditis elegans. EMBO J. EMBO Press; 2011;30: 1376–88. 10.1038/emboj.2011.13 21304490 PMC3094115

[50] Chalasani SH, Chronis N, Tsunozaki M, Gray JM, Ramot D, Goodman MB, et al Dissecting a circuit for olfactory behaviour in Caenorhabditis elegans. Nature. Nature Publishing Group; 2007;450: 63–70. 10.1038/nature06292 17972877

[51] Rose JK, Kaun KR, Rankin CH. A new group-training procedure for habituation demonstrates that presynaptic glutamate release contributes to long-term memory in Caenorhabditis elegans. Learn Mem. 2002;9: 130–7. 10.1101/lm.46802 12075001 PMC182588

[52] Keane J, Avery L. Mechanosensory Inputs Influence Caenorhabditis elegans Pharyngeal Activity via Ivermectin Sensitivity Genes. Genetics. 2003;164: 153–162. Available: http://www.genetics.org/content/164/1/153.short 12750328 10.1093/genetics/164.1.153 PMC1462566

[53] Cook A, Aptel N, Portillo V, Siney E, Sihota R, Holden-Dye L, et al Caenorhabditis elegans ivermectin receptors regulate locomotor behaviour and are functional orthologues of Haemonchus contortus receptors. Mol Biochem Parasitol. 2006;147: 118–25. 10.1016/j.molbiopara.2006.02.003 16527366

[54] Rufener L, Bedoni N, Baur R, Rey S, Glauser DA, Bouvier J, et al acr-23 Encodes a monepantel-sensitive channel in Caenorhabditis elegans. PLoS Pathog. Public Library of Science; 2013;9: e1003524 10.1371/journal.ppat.1003524 23950710 PMC3738477

[55] Lewis J, Wu C, Levine J, Berg H. Levamisole-resitant mutants of the nematode Caenorhabditis elegans appear to lack pharmacological acetylcholine receptors. Neuroscience. 1980;5: 967–989. 10.1016/0306-4522(80)90180-3 7402460

[56] Buxton SK, Charvet CL, Neveu C, Cabaret J, Cortet J, Peineau N, et al Investigation of acetylcholine receptor diversity in a nematode parasite leads to characterization of tribendimidine- and derquantel-sensitive nAChRs. PLoS Pathog. 2014;10: e1003870 10.1371/journal.ppat.1003870 24497826 PMC3907359

[57] Robertson AP, Clark CL, Burns TA, Thompson DP, Geary TG, Trailovic SM, et al Paraherquamide and 2-deoxy-paraherquamide distinguish cholinergic receptor subtypes in Ascaris muscle. J Pharmacol Exp Ther. 2002;302: 853–60. 10.1124/jpet.102.034272 12183640

[58] Parkinson J, Mitreva M, Whitton C, Thomson M, Daub J, Martin J, et al A transcriptomic analysis of the phylum Nematoda. Nat Genet. 2004;36: 1259–67. 10.1038/ng1472 15543149

[59] Freeman AS, Nghiem C, Li J, Ashton FT, Guerrero J, Shoop WL, et al Amphidial structure of ivermectin-resistant and susceptible laboratory and field strains of Haemonchus contortus. Vet Parasitol. 2003;110: 217–226. 12482650 10.1016/s0304-4017(02)00321-7

[60] Angstadt JD, Donmoyer JE, Stretton AO. Retrovesicular ganglion of the nematode Ascaris. J Comp Neurol. 1989;284: 374–88. 10.1002/cne.902840305 2754041

[61] Davis RE, Stretton AO. Extracellular recordings from the motor nervous system of the nematode, Ascaris suum. J Comp Physiol A. 1992;171: 17–28. Available: http://www.ncbi.nlm.nih.gov/pubmed/1328624 1328624 10.1007/BF00195957

[62] Stretton AO, Fishpool RM, Southgate E, Donmoyer JE, Walrond JP, Moses JE, et al Structure and physiological activity of the motoneurons of the nematode Ascaris. Proc Natl Acad Sci. 1978;75: 3493–3497. 10.1073/pnas.75.7.3493 277952 PMC392804

[63] Portillo V, Jagannathan S, Wolstenholme AJ. Distribution of glutamate-gated chloride channel subunits in the parasitic nematode Haemonchus contortus. J Comp Neurol. 2003;462: 213–22. 10.1002/cne.10735 12794744

[64] Glendinning SK, Buckingham SD, Sattelle DB, Wonnacott S, Wolstenholme AJ. Glutamate-gated chloride channels of Haemonchus contortus restore drug sensitivity to ivermectin resistant Caenorhabditis elegans. Carlow CKS, editor. PLoS One. Public Library of Science; 2011;6: e22390 10.1371/journal.pone.0022390 21818319 PMC3144221

[65] Geary TG, Sims SM, Thomas EM, Vanover L, Davis JP, Winterrowd C, et al Haemonchus contortus: ivermectin-induced paralysis of the pharynx. Exp Parasitol. 1993;77: 88–96. 10.1006/expr.1993.1064 8344410

[66] Avery L, Horvitz HR. Pharyngeal pumping continues after laser killing of the pharyngeal nervous system of C. elegans. Neuron. 1989;3: 473–485. 10.1016/0896-6273(89)90206-7 2642006

[67] Paiement JP, Leger C, Ribeiro P, Prichard RK. Haemonchus contortus: effects of glutamate, ivermectin, and moxidectin on inulin uptake activity in unselected and ivermectin-selected adults. Exp Parasitol. 1999;92: 193–198. 10.1006/expr.1999.4413 10403760

[68] Bennett JL, Williams JF, Dave V. Pharmacology of ivermectin. Parasitol Today. 1988;4: 226–228. 10.1016/0169-4758(88)90163-9 15463103

[69] Tompkins JB, Stitt LE, Ardelli BF. Brugia malayi: In vitro effects of ivermectin and moxidectin on adults and microfilariae. Exp Parasitol. Elsevier Inc.; 2010;124: 394–402. 10.1016/j.exppara.2009.12.003 20034492

[70] Lok JB, Pollack RJ, Donnelly JJ. Studies of the growth-regulating effects of ivermectin on larval Onchocerca lienalis in vitro. J Parasitol. 1987;73: 80–84. Available: http://www.ncbi.nlm.nih.gov/pubmed/3572669 3572669

[71] Moreno Y, Nabhan JF, Solomon J, Mackenzie CD, Geary TG. Ivermectin disrupts the function of the excretory-secretory apparatus in microfilariae of Brugia malayi. Proc Natl Acad Sci U S A. 2010;107: 20120–5. 10.1073/pnas.1011983107 21041637 PMC2993382

[72] Li BW, Rush AC, Weil GJ. High level expression of a glutamate-gated chloride channel gene in reproductive tissues of Brugia malayi may explain the sterilizing effect of ivermectin on filarial worms. Int J Parasitol Drugs Drug Resist. Australian Society for Parasitology; 2014;4: 71–6. 10.1016/j.ijpddr.2014.01.002 25057456 PMC4095040

[73] McGarry HF, Plant LD, Taylor MJ. Diethylcarbamazine activity against Brugia malayi microfilariae is dependent on inducible nitric-oxide synthase and the cyclooxygenase pathway. Filaria J. 2005;4: 4 10.1186/1475-2883-4-4 15932636 PMC1173132

[74] Goodman MB, Hall DH, Avery L, Lockery SR. Active Currents Regulate Sensitivity and Dynamic Range in C. elegans Neurons. Neuron. Elsevier; 1998;20: 763–772. 10.1016/S0896-6273(00)81014-4 9581767 PMC4444786

[75] Friedland AE, Tzur YB, Esvelt KM, Colaiácovo MP, Church GM, Calarco JA. Heritable genome editing in C. elegans via a CRISPR-Cas9 system. Nat Methods. Nature Publishing Group, a division of Macmillan Publishers Limited. All Rights Reserved.; 2013;10: 741–3. 10.1038/nmeth.2532 23817069 PMC3822328

[76] Frøkjaer-Jensen C, Davis MW, Hopkins CE, Newman BJ, Thummel JM, Olesen S-P, et al Single-copy insertion of transgenes in Caenorhabditis elegans. Nat Genet. Nature Publishing Group; 2008;40: 1375–83. 10.1038/ng.248 18953339 PMC2749959

[77] Chiu H, Schwartz HT, Antoshechkin I, Sternberg PW. Transgene-Free Genome Editing in Caenorhabditis elegans Using CRISPR-Cas. Genetics. 2013;195: 1167–1171. 10.1534/genetics.113.155879 23979577 PMC3813845

[78] Lo T-W, Pickle CS, Lin S, Ralston EJ, Gurling M, Schartner CM, et al Precise and Heritable Genome Editing in Evolutionarily Diverse Nematodes Using TALENs and CRISPR/Cas9 to Engineer Insertions and Deletions. Genetics. 2013;195: 331–348. 10.1534/genetics.113.155382 23934893 PMC3781963

[79] Waaijers S, Portegijs V, Kerver J, Lemmens BBCG, Heuvel S Van Den, Boxem M, et al CRISPR/Cas9-Targeted Mutagenesis in Caenorhabditis elegans. Genetics. 2013;195: 1187–1191. 10.1534/genetics.113.156299 23979586 PMC3813849

[80] Tzur YB, Friedland AE, Nadarajan S, Church GM, Calarco JA, Colaiácovo MP. Heritable Custom Genomic Modifications in Caenorhabditis elegans via a CRISPR-Cas9 System. Genetics. 2013;195: 1181–1185. 10.1534/genetics.113.156075 23979579 PMC3813848

[81] Noda M, Takahashi H, Tanabe T, Toyosato M, Kikyotani S, Furutani Y, et al Structural homology of Torpedo californica acetylcholine receptor subunits. Nature. Nature Publishing Group; 1983;302: 528–532. 10.1038/302528a0 6188060

