# Supplementary material for: The Validation of Nematode-Specific Acetylcholine-Gated Chloride Channels as Potential Anthelmintic Drug Targets
Source: PLoS One. 2015 Sep 22;10(9):e0138804. doi: 10.1371/journal.pone.0138804 (PMC4578888; doi:10.1371/journal.pone.0138804)
Supplement: S1 Fig — Depiction of the deleted genomic regions in the strains vc1757 (Δacc-2), vc40013 (Δlgc-49), rb2187 (Δlgc-47), and tm3268 (Δacc-1). (DOCX) [file pone.0138804.s001.docx]

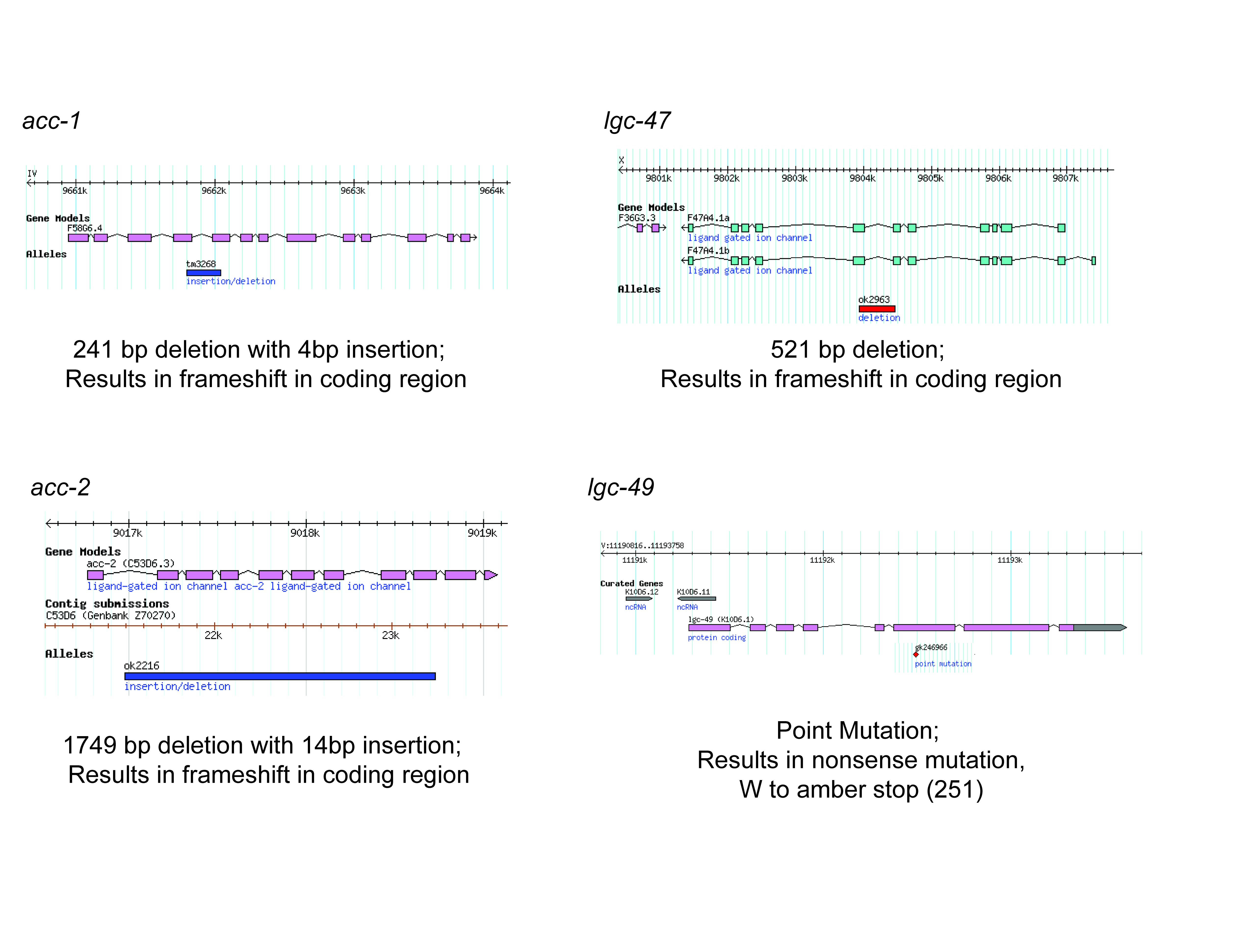


S1 Fig: ACC mutant alleles

Depiction of alleles used for the investigation of *acc* mutant phenotypes. All information in this figure was obtained on wormbase.org.
