# Supplementary material for: The Validation of Nematode-Specific Acetylcholine-Gated Chloride Channels as Potential Anthelmintic Drug Targets
Source: PLoS One. 2015 Sep 22;10(9):e0138804. doi: 10.1371/journal.pone.0138804 (PMC4578888; doi:10.1371/journal.pone.0138804)
Supplement: S2 Fig — Worms with AVR-15::YFP expressed under control of acc-2, acc-3, lgc-47, and lgc-49-promoters fail to develop on 50ng/mL IVM. Worms with AVR-15::YFP expressed under control of lgc-48-promoter develop to adulthood, while worms with AVR-15::YFP expressed exclusively in ACC-1-expressing tissues exhibit delayed growth on 50ng/mL IVM, but do reach adulthood. (DOCX) [file pone.0138804.s002.docx]

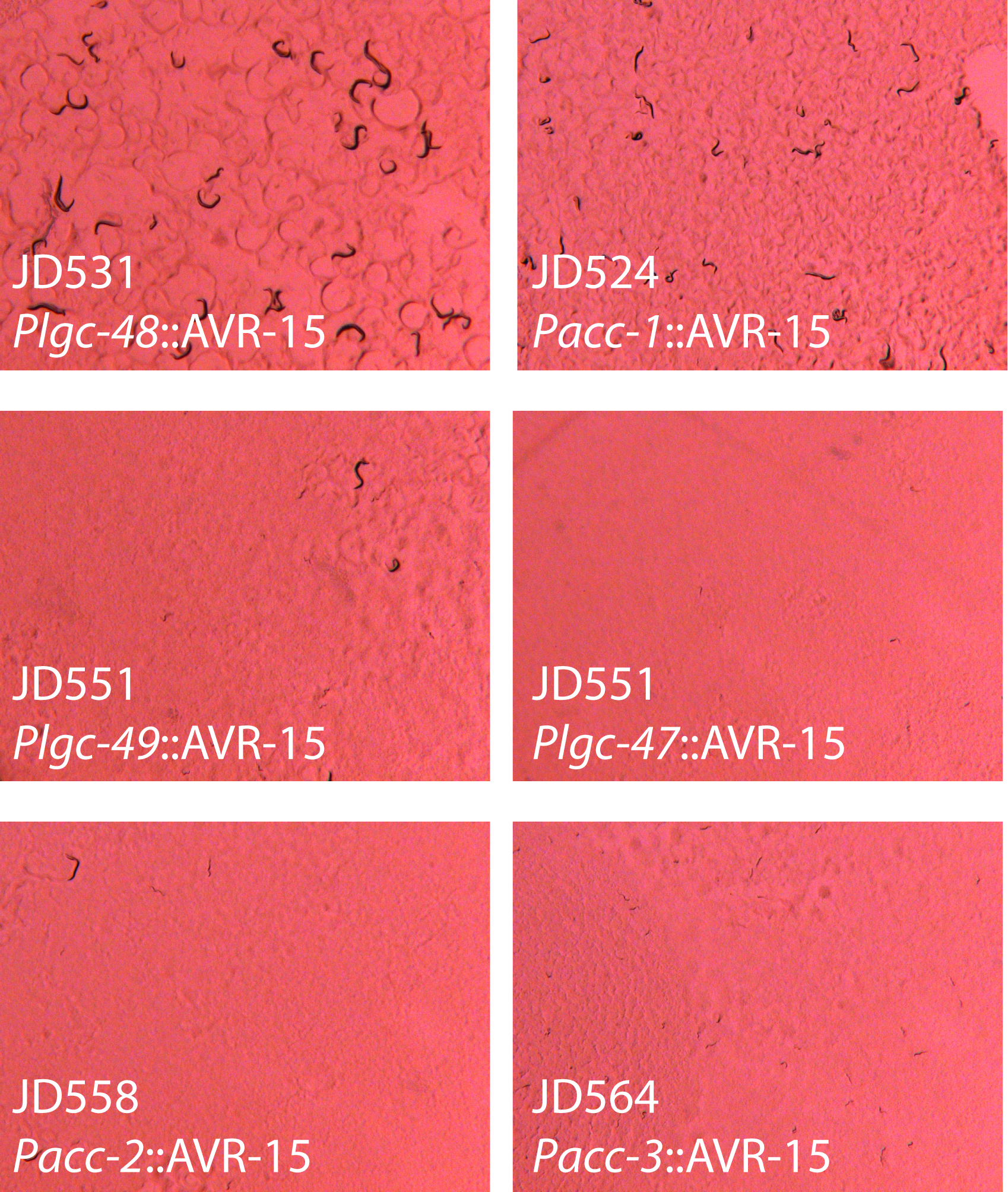


S2 Fig: Growth of strains on 50ng/mL IVM on day three

Worms with AVR-15::YFP expressed under control of *acc-2, acc-3, lgc-47,* and *lgc-49-*promoters fail to develop on 50ng/mL IVM. Worms with AVR-15::YFP expressed under control of *lgc-48*-promoter develop to adulthood, while worms with AVR-15::YFP expressed exclusively in ACC-1-expressing tissues exhibit delayed growth on 50ng/mL IVM, but do reach adulthood.
