# Supplementary material for: The Validation of Nematode-Specific Acetylcholine-Gated Chloride Channels as Potential Anthelmintic Drug Targets
Source: PLoS One. 2015 Sep 22;10(9):e0138804. doi: 10.1371/journal.pone.0138804 (PMC4578888; doi:10.1371/journal.pone.0138804)
Supplement: S1 Table — Table listing all genes used in constructing the ACC phylogeny (Fig 1). Included for each gene is the corresponding GI accession number, GenBank accession number, the original name assigned to that gene, the organism in which that gene was found, and the new ACC name we have assigned to the gene. (DOCX) [file pone.0138804.s003.docx]

S1 Table: ACC Orthologs

| GI Number | GenBank Accession | Original Name | Organism | New Name |
| --- | --- | --- | --- | --- |
| 597837315 | EYB86945.1 | hypothetical protein Y032_0271g909 | Ancylostoma ceylanicum | Ace-ACC-1 |
| 597863198 | EYC12603.1 | hypothetical protein Y032_0046g1320 | Ancylostoma ceylanicum | Ace-LGC-49 |
| 597868843 | EYC18229.1 | hypothetical protein Y032_0028g1747 | Ancylostoma ceylanicum | Ace-LGC-47 |
| 597880510 | EYC29873.1 | hypothetical protein Y032_0005g2309 | Ancylostoma ceylanicum | Ace-ACC-2 |
| 597866827 | EYC16220.1 | hypothetical protein Y032_0034g2868 | Ancylostoma ceylanicum | Ace-ACC-3 |
| 597845895 | EYB95414.1 | hypothetical protein Y032_0160g3339 | Ancylostoma ceylanicum | Ace-LGC-46 |
| 597867797 | EYC17187.1 | hypothetical protein Y032_0031g2323 | Ancylostoma ceylanicum | Ace-ACC-4 |
| 597872674 | EYC22050.1 | hypothetical protein Y032_0018g3684 | Ancylostoma ceylanicum | Ace-LGC-48 |
| 541041726 | ERG80946.1 | ligand-gated ion channel 50 | Ascaris suum | Asu-ACC-3 |
| 170589049 |  | Neurotransmitter-gated ion-channel ligand binding domain containing protein | Brugia malayi | Bma-LGC-47 |
| 170591178 |  | Neurotransmitter-gated ion-channel ligand binding domain containing protein | Brugia malayi | Bma-LGC-46 |
| 671418947 | CDQ04816.1 | Protein BM-ACC-4, isoform c | Brugia malayi | Bma-ACC-4 |
| 560133579 | CDJ86191.1 | unnamed protein product, partial | Haemonchus contortus | Hco-ACC-1 |
| 560121722 | CDJ93640.1 | unnamed protein product, partial | Haemonchus contortus | Hco-LGC-47 |
| 560139349 | CDJ82184.1 | unnamed protein product | Haemonchus contortus | Hco-ACC-2 |
| 560120919 | CDJ94449.1 | unnamed protein product, partial | Haemonchus contortus | Hco-ACC-3 |
| 560140641 | CDJ80925.1 | unnamed protein product | Haemonchus contortus | Hco-LGC-46 |
| 560121098 | CDJ94263.1 | unnamed protein product, partial | Haemonchus contortus | Hco-ACC-4 |
| 312104287 |  | ACC-1 protein, partial | Loa loa | Llo-ACC-1 |
| 393910956 | EJD76102.1 | ACC-1 protein, variant | Loa loa | Llo-LGC-47 |
| 393911884 | EJD76487.1 | gamma-aminobutyric acid receptor subunit beta | Loa loa | Llo-ACC-5 |
| 393910497 | EFO21764.2 | hypothetical protein LOAG_06725 | Loa loa | Llo-ACC-3 |
| 393910899 | EFO21304.2 | hypothetical protein LOAG_07182 | Loa loa | Llo-ACC-4 |
| 312086659 |  | hypothetical protein LOAG_09589 | Loa loa | Llo-LGC-46 |
| 568269520 | ETN70865.1 | Neurotransmitter-gated ion-channel ligand binding domain protein | Necator americanus | Nam-LGC-49 |
| 568286953 | ETN75650.1 | Neurotransmitter-gated ion-channel ligand binding domain protein | Necator americanus | Nam-ACC-2 |
| 568288576 | ETN76901.1 | Neurotransmitter-gated ion-channel ligand binding domain protein | Necator americanus | Nam-ACC-3 |
| 505858599 |  | PREDICTED: ligand-gated ion channel 50-like, partial | Sorex araneus | Soa-ACC-6 |
| 685824230 | CEF59284.1 | Gamma-aminobutyric acid A receptor/Glycine receptor alpha family and Neurotransmitter-gated ion-channel transmembrane domain and Neurotransmitter-gated ion-channel family and Neurotransmitter-gated ion-channel ligand-binding domain-containing protein 7 | Strongyloides ratti | Sra-ACC-1 |
| 685825353 | CEF60405.1 | Gamma-aminobutyric acid A receptor/Glycine receptor alpha family and Neurotransmitter-gated ion-channel transmembrane domain and Neurotransmitter-gated ion-channel family and Neurotransmitter-gated ion-channel ligand-binding domain-containing protein 2 | Strongyloides ratti | Sra-LGC-47 |
| 685826098 | CEF61143.1 | Gamma-aminobutyric acid A receptor/Glycine receptor alpha family and Neurotransmitter-gated ion-channel transmembrane domain and Neurotransmitter-gated ion-channel family and Neurotransmitter-gated ion-channel ligand-binding domain-containing protein 27 | Strongyloides ratti | Sra-ACC-5 |
| 685834435 | CEF69356.1 | Gamma-aminobutyric acid A receptor/Glycine receptor alpha family and Neurotransmitter-gated ion-channel transmembrane domain and Neurotransmitter-gated ion-channel family and Neurotransmitter-gated ion-channel ligand-binding domain-containing protein 11 | Strongyloides ratti | Sra-ACC-3 |
| 685836452 | CEF71372.1 | Gamma-aminobutyric acid A receptor/Glycine receptor alpha family and Neurotransmitter-gated ion-channel transmembrane domain and Neurotransmitter-gated ion-channel family and Neurotransmitter-gated ion-channel ligand-binding domain-containing protein 8 | Strongyloides ratti | Sra-ACC-4 |
| 685831296 | CEF66217.1 | Gamma-aminobutyric acid A receptor/Glycine receptor alpha family and Neurotransmitter-gated ion-channel transmembrane domain and Neurotransmitter-gated ion-channel family and Neurotransmitter-gated ion-channel ligand-binding domain-containing protein 18 | Strongyloides ratti | Sra-LGC-46 |
| 339233638 |  | putative glycine receptor subunit alpha-3 | Trichinella spiralis | Tsp-ACC-7 |
| 339238051 |  | gamma-aminobutyric acid receptor subunit theta | Trichinella spiralis | Tsp-ACC-6 |
| 339241167 |  | glycine receptor subunit beta-type 4 | Trichinella spiralis | Tsp-LGC-46 |
| 339251582 |  | putative neurotransmitter-gated ion-channel ligand binding domain protein | Trichinella spiralis | Tsp-ACC-4 |
| 669332095 | KFD72262.1 | hypothetical protein M514_01324 | Trichuris suis | Tsu-ACC-7 |
| 669311843 | KFD55310.1 | hypothetical protein M513_03951 | Trichuris suis | Tsu-ACC-6 |
| 669331006 | KFD71178.1 | hypothetical protein M514_04974 | Trichuris suis | Tsu-LGC-46 |
| 669333012 | KFD73175.1 | hypothetical protein M514_04811 | Trichuris suis | Tsu-ACC-4 |
| 669226709 | CDW52008.1 | protein lgc; protein cbr acc-1; protein acc; cre lgc-49 protein; cre acc-1 protein; cbn acc-1 protein | Trichuris trichiura | Ttr-ACC-7 |
| 669221264 | CDW57435.1 | protein lgc; protein cbr lgc-49; protein cbr acc-2; protein acc; cre lgc-49 protein; cbn acc-3 protein | Trichuris trichiura | Ttr-ACC-6 |
| 669226778 | CDW51961.1 | Neur chan LBD and Neur chan memb domain containing protein | Trichuris trichiura | Ttr-LGC-46 |
| 669225492 | CDW53217.1 | Ligand gated ion channel 50 | Trichuris trichiura | Ttr-ACC-4 |
| 402592951 | EJW86878.1 | acetylcholine-gated chloride channel subunit ACC-3 | Wuchereria bancrofti | Wba-LGC-47 |
| 402592452 | EJW86381.1 | hypothetical protein WUBG_02706 | Wuchereria bancrofti | Wba-LGC-46 |
| 402592399 | EJW86328.1 | hypothetical protein WUBG_02761, partial | Wuchereria bancrofti | Wba-ACC-3 |
| 402585513 | EJW79453.1 | hypothetical protein WUBG_09639 | Wuchereria bancrofti | Wba-ACC-4 |

S1 Table: ACC Orthologs, continued
