# Supplementary material for: The Validation of Nematode-Specific Acetylcholine-Gated Chloride Channels as Potential Anthelmintic Drug Targets
Source: PLoS One. 2015 Sep 22;10(9):e0138804. doi: 10.1371/journal.pone.0138804 (PMC4578888; doi:10.1371/journal.pone.0138804)
Supplement: S2 Table — Table describing all primers used to clone all the acc promoters used in the acc::AVR-15 constructs. Gene-specific sequence in lowerase, vector-specific sequence in uppercase. (DOCX) [file pone.0138804.s004.docx]

| Primer Description | Primer Sequence (5’-3’) |
| --- | --- |
| LGC-49 (K10D6.1) Promoter Forward Primer | CGACTCTAGAGGATCCtcatttgtcatccgtgacgt |
| LGC-49 (K10D6.1) Promoter Reverse Primer | GGATGAGACAGCTGGCCAgatagctggttgtcgagt |
| LGC-47 (F47A4.1) Promoter Forward Primer | CGACTCTAGAGGATCCgcccagccttctcagcacttat |
| LGC-47 (F47A4.1) Promoter Reverse Primer | GGATGAGACAGCTGGCCAatcacacaagggcccgt |
| LGC-48 (C50B6.11) Promoter Forward Primer | CGACTCTAGAGGATCCacactcccgttggaatcc |
| LGC-48 (C50B6.11) Promoter Reverse Primer | GGATGAGACAGCTGGCCAttagcgtcaagttggctttac |
| ACC-1 Promoter Forward Primer | CGACTCTAGAGGATCCttgcctccttccattgcctttg |
| ACC-1 Promoter Reverse Primer | GGATGAGACAGCTGGCCActacagtaagctcatcccacca |
| ACC-2 Promoter Forward Primer | CGACTCTAGAGGATCCtcgctgcaggccttcagaa |
| ACC-2 Promoter Reverse Primer | GGATGAGACAGCTGGCCAcatgaagctcgtcccacatt |
| ACC-3 Promoter Forward Primer | CGACTCTAGAGGATCCtgtcgttgtcgttgtcgtc |
| ACC-3 Promoter Forward Primer | GGATGAGACAGCTGGCCAatttgtgggaaacggagttg |

S2 Table: Primers used for ACC promoter cloning

(Gene-specific sequence in lowerase, vector-specific sequence in uppercase)
