# Supplementary material for: The Validation of Nematode-Specific Acetylcholine-Gated Chloride Channels as Potential Anthelmintic Drug Targets
Source: PLoS One. 2015 Sep 22;10(9):e0138804. doi: 10.1371/journal.pone.0138804 (PMC4578888; doi:10.1371/journal.pone.0138804)
Supplement: S3 Table — Primers used to screen for deletions in strains vc1757 (Δacc-2), vc40013 (Δlgc-49), rb2187 (Δlgc-47), and tm3268 (Δacc-1). (DOCX) [file pone.0138804.s005.docx]

| Primer Description | Primer Sequence (5’-3’) |
| --- | --- |
| VC1757 inner 5’ | TCTCTCACTTCCGCTGACCT |
| VC1757 inner 3’ | TTCTTTCAAACCAAACGGGTC |
| VC40013 mut scr for | ATCCACGCGAATTTCGGCAC |
| VC40013 mut scr rev | GTTGGGAGATATGCTTGCATG |
| RB2187 inner left | TCCTTTCATTCTTTTGCTCACA |
| RB2187 inner right | AAGCGGAAAGTGTTTCTCCTC |
| TM3268_inner_5 | CCGGGTTGGATTATGGTGTC |
| TM3268_inner_3 | GCTCGTCCCACATTCCAGCT |

S3 Table: Primers used to screen for deletions in strains vc1757 (*Δacc-2*), vc40013 (Δ*lgc-49)*, rb2187 (Δ*lgc-47),* and tm3268 (*Δacc-1*).
