# Supplementary material for: The Validation of Nematode-Specific Acetylcholine-Gated Chloride Channels as Potential Anthelmintic Drug Targets
Source: PLoS One. 2015 Sep 22;10(9):e0138804. doi: 10.1371/journal.pone.0138804 (PMC4578888; doi:10.1371/journal.pone.0138804)
Supplement: S4 Table — Table listing all the worm strains used, including a list of the alleles each strain is carrying. (DOCX) [file pone.0138804.s006.docx]

| Strain Name | Alleles |
| --- | --- |
| JD369 | avr-14(vu47) I; glc-3(ok321) avr-15(vu227) glc-1(pk54) V |
| JD524 | avr-14(vu47) I; glc-3(ok321) avr-15(vu227) glc-1(pk54) V; vuIs231 [pPD49.26 Pacc-1::AVR-15::YFP, pRAK] |
| JD526 | avr-14(vu47) I; glc-3(ok321) avr-15(vu227) glc-1(pk54) V; vuIs231 [pPD49.26 Pacc-1::AVR-15::YFP, pRAK] |
| JD530 | avr-14(vu47) I; glc-3(ok321) avr-15(vu227) glc-1(pk54) V; vuIs237 [pPD49.26 Plgc-48::AVR-15::YFP, pRAK] |
| JD531 | avr-14(vu47) I; glc-3(ok321) avr-15(vu227) glc-1(pk54) V; vuIs238 [pPD49.26 Plgc-48::AVR-15::YFP, pRAK] |
| JD554 | avr-14(vu47) I; glc-3(ok321) avr-15(vu227) glc-1(pk54) V; vuIs254 [pPD49.26 pF47A4.1::AVR-15::YFP, pRAK] |
| JD555 | avr-14(vu47) I; glc-3(ok321) avr-15(vu227) glc-1(pk54) V; vuIs255 [pPD49.26 pF47A4.1::AVR-15::YFP, pRAK] |
| JD558 | avr-14(vu47) I; glc-3(ok321) avr-15(vu227) glc-1(pk54) V; vuIs258 [pPD49.26 pACC-2::AVR-15::YFP, pRAK] |
| JD562 | avr-14(vu47) I; glc-3(ok321) avr-15(vu227) glc-1(pk54) V; vuIs262 [pPD49.26 pACC-2::AVR-15::YFP, pRAK] |
| JD564 | avr-14(vu47) I; glc-3(ok321) avr-15(vu227) glc-1(pk54) V; vuIs264 [pPD49.26 pACC-3::AVR-15::YFP, pRAK] |
| JD565 | avr-14(vu47) I; glc-3(ok321) avr-15(vu227) glc-1(pk54) V; vuIs265 [pPD49.26 pACC-3::AVR-15::YFP, pRAK] |
| JD639 | avr-14(vu47) I; glc-3(ok321) avr-15(vu227) glc-1(pk54) V; vuIs297 [pPD49.26 plgc-49::AVR-15::YFP, pRAK] |
| JD640 | avr-14(vu47) I; glc-3(ok321) avr-15(vu227) glc-1(pk54) V; vuIs298 [pPD49.26 plgc-49::AVR-15::YFP, pRAK] |
| TM3268 | acc-1 (tm3268) |
| VC1757 | acc-2 (ok2216) |
| RB2187 | lgc-47 (ok2963) |
| VC40013 | lgc-49 (gk246966) |

S4 Table: Worm Strains Used
